# Multisource noninvasive genetics of brown bears (*Ursus arctos*) in Greece reveals a highly structured population and a new matrilineal contact zone in southern Europe

**DOI:** 10.1002/ece3.7493

**Published:** 2021-05-02

**Authors:** Charilaos Pylidis, Peeter Anijalg, Urmas Saarma, Deborah A. Dawson, Nikoleta Karaiskou, Roger Butlin, Yorgos Mertzanis, Alexios Giannakopoulos, Yorgos Iliopoulos, Andrew Krupa, Terence A. Burke

**Affiliations:** ^1^ School of Biological Sciences University of Bristol Bristol UK; ^2^ NERC Biomolecular Analysis Facility Department of Animal and Plant Sciences University of Sheffield UK; ^3^ Callisto Wildlife and Nature Conservation Society Thessaloniki Greece; ^4^ Department of Zoology Institute of Ecology and Earth Sciences University of Tartu Tartu Estonia; ^5^ Department of Genetics, Developmental and Molecular Biology School of Biology Aristotle University of Thessaloniki Thessaloniki Greece; ^6^ Department of Animal and Plant Sciences University of Sheffield Sheffield UK

**Keywords:** connectivity, contact zone, Greece, phylogeography, population size, population structure, rear‐edges, *Ursus arctos*

## Abstract

In human‐dominated landscapes, connectivity is crucial for maintaining demographically stable mammalian populations. Here, we provide a comprehensive noninvasive genetic study for the brown bear population in the Hellenic Peninsula. We analyze its population structuring and connectivity, estimate its population size throughout its distribution, and describe its phylogeography in detail for the first time. Our results, based on 150 multilocus genotypes and on 244‐bp sequences of the mtDNA control region, show the population is comprised by three highly differentiated genetic clusters, consistent with geographical populations of Pindos, Peristeri, and Rhodope. By detecting two male bears with Rhodopean ancestry in the western demes, we provide strong evidence for the ongoing genetic connectivity of the geographically fragmented eastern and western distributions, which suggests connectivity of the larger East Balkan and Pindos‐Dinara populations. Total effective population size (*N*_e_) was estimated to be 199 individuals, and total combined population size (*N*_C_) was 499, with each cluster showing a relatively high level of genetic variability, suggesting that migration has been sufficient to counteract genetic erosion. The mtNDA results were congruent with the microsatellite data, and the three genetic clusters were matched predominantly with an equal number of mtDNA haplotypes that belong to the brown bear Western mitochondrial lineage (Clade 1), with two haplotypes being globally new and endemic. The detection of a fourth haplotype that belongs to the Eastern lineage (Clade 3a1) in three bears from the western distribution places the southernmost secondary contact zone between the Eastern and Western lineages in Greece and generates new hypotheses about postglacial maxima migration routes. This work indicates that the genetic composition and diversity of Europe's low‐latitude fringe population are the outcome of ancient and historical events and highlight its importance for the connectivity and long‐term persistence of the species in the Balkans.

## Graphical abstract

Using microsatellites and mtDNA, we reveal the genetic structure, diversity, population size of the brown bear in Greece, and its detailed phylogeography for the first time, revealing a new matrilinear contact zone

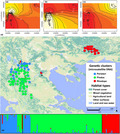

## 1 INTRODUCTION

The genetic patterns of extant biota are the result of ancient natural processes as well as historical and contemporary events. Events, such as population size depletion, range shifts, and bottlenecks, have undoubtedly shaped the distribution of genetic variation of European wildlife (Hewitt, 1999). One group of animals that have been a subject to dramatic biogeographical shifts over the past millennia are large carnivores. They are a particularly controversial group which have large spatial requirements, and though elusive by nature, the damages they inflict on husbandry, agriculture, and property do not go unnoticed. Conflict with humans in combination with profound habitat alteration over the past centuries has been the major driving force for their extirpation throughout the Northern Hemisphere (Ripple et al., 2014). Yet, despite being driven to extinction by extermination in many parts of their historical range, they persevered by retreating to remote and inaccessible to humans areas. In the 21st century, their decline has been somewhat halted in Europe and their recovery is evident in many regions (Chapron et al., 2014).

**FIGURE 1:**
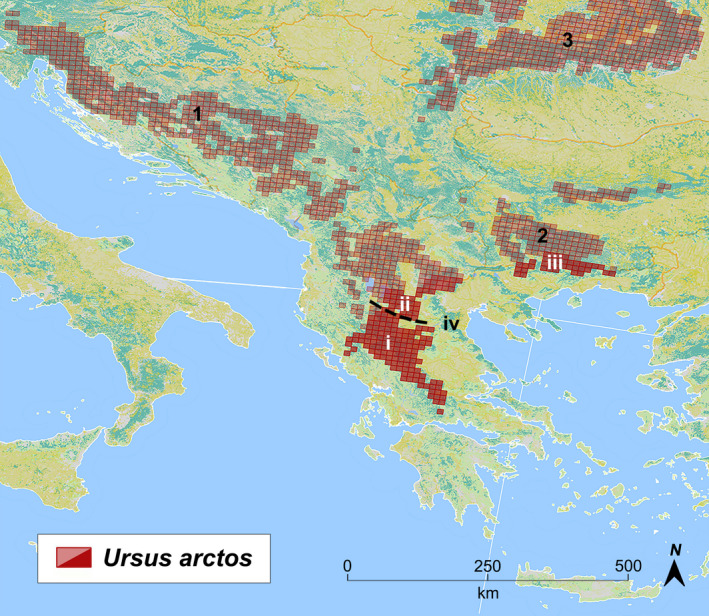
Brown bears in Greece (saturated grids) in the (i) Pindos, (ii) Peristeri mountain ranges with the dashed line representing the geographical boundary between the two, and (iii) in Rhodope, which form the tips of the larger Pindos‐Dinara and East Balkan distribution (1 and 2, respectively). The locations of Mount Olympus (iv) and the Carpathian population (3) have also been depicted

Contraction–expansion events are expected to have profound effects on the genetic diversity of a population and on its evolutionary potential (Arenas et al., 2012). The contemporary distribution of Europe's largest extant carnivore the brown bear (*Ursus arctos* L., 1758) was primarily shaped by the Late Pleistocene glaciations when bears primarily took refuge in the Mediterranean Peninsulas and most likely also in the Carpathian–Caucasus and some unknown refugia in the east, from where the species recolonized the continent, reaching as far north as Scandinavia (Anijalg et al., 2018; Davison et al., 2011; Hewitt, 2000; Saarma et al., 2007; Taberlet & Bouvet, 1994). Historically, the brown bear in Europe has been distributed throughout forested habitats and mountainous terrains. In recent centuries, human‐driven extermination and reduction events have reduced their population and the once abundant ursid throughout Europe now inhabits a fraction of its former range (Servheen et al., 1999). Since the 1930s, the combined effects of legislative measures and conservation efforts have helped brown bears to make an astounding recovery, especially in Northern Europe (Swenson et al., 1995). Widespread populations are now found in Fennoscandia, Russia, in the Carpathians, and in the Northern Balkans, while in southern latitudes, the Mediterranean and Balkan Peninsulas are occupied by smaller fragmented populations (Davison et al., 2011; Kaczensky et al. 2012). From a geographical perspective, the low‐latitude fringe of their European range is found in mainland Greece (Figure 1). Traditionally, its geographical description has been dividing the population into two demes, Pindos in the western and Rhodope eastern part of the mainland, which form the tips of the larger Dinaric‐Pindos and East Balkan distributions, respectively (Mertzanis, 1999; Mertzanis et al. 2009). Pindos and Rhodope are thought to be genetically disconnected (Karamanlidis, 2011), and the former is comprised by two geographical populations named Peristeri and the homonym Pindos (i & ii in Figure 1, respectively) to reflect the respective mountainous regions which dominate the landscape. In the north, Peristeri is part of the elevated crest that extends in from Mt Varnous (Baba Mt) in North Macedonia. In the south, Pindos, the largest of the two, is formed by the vast ridge that extends from mount Grammos in the border with Albania extending all the way to central Greece and dominates the landscape.

Historical evidence suggests that the brown bear has been present across mainland Northern Greece since the last glacial maximum (Mertzanis, 1999). It is postulated that bears persisted in the Peloponnese Peninsula (N37.6°, E22.3°) until the 15th–16th century, following a dramatic human‐driven population reduction, when bears were hunted for their skin, a common practice around Europe at the time (Enserink & Vogel, 2006). It is also thought that the geographical split between the eastern and western demes occurred around that time. By the 1960s, the bear population occupied only a fraction of its historical range, with bears receding into the rugged mountainous landscape which provided suitable habitat and remoteness from human disturbance (Mertzanis, 1999). The decline of the bear population continued until the end of the 20th century and when legislation enforcement, rural abandonment, the establishment of a compensation system, and increasing social acceptance, were the catalysts for reversing its negative trend, aiding its gradual recovery. Recently, the bears of the Hellenic Peninsula started to reclaim areas of their historical range as far as the 38th parallel and for the first time in 60 years, bears have roaming again in natural landmarks such as Mount Olympus (N40.08°, E22.35°).

Small and isolated populations that have undergone demographic crashes are expected to show lower genetic diversity than larger populations due to drift and restricted gene flow. In addition, marginal populations such as the brown bears of Greece may be particularly vulnerable to contraction–expansion events, especially in the presence of biogeographical and anthropogenic barriers. This is particularly important as southern margin populations of temperate species in former glacial refugia became the “rear‐edges,” the low‐latitude fringes of large continuous distributions which are recognized as long‐term stores of adaptive genetic diversity (Hampe & Petit, 2005; Petit et al., 2003).

The distribution of the bear population in the Hellenic peninsula provides the opportunity to study the southern fringe of its European range and to assess the connectivity of the fragmented Balkan distribution. In recent years, the connectivity between distant bear populations in Northern Europe has been revealed (Kopatz et al., 2014; Tammeleht et al., 2010) yet such information is lacking for southern Europe. Understanding the genetic patterns of a population that has persisted since the LGM and in a human‐dominated landscape requires looking into the recent and historical processes which have shaped its demographic history. Measuring differentiation, estimating connectivity and genetic diversity, and producing robust demographic parameters are important to assess the status of small and fragmented populations and for designing management actions targeting their conservation. To get a comprehensive picture of both contemporary and past gene flow dynamics of continental Europe's largest carnivore in its low‐latitude margin, we utilized both microsatellites and mtDNA. Using noninvasive genetic methods, we (a) tested for population genetic structure within, and differentiation between the eastern and western bear populations, (b) assessed the diversity of, the connectivity and migration between the two regions, (c) estimated total and effective population size while testing for signatures of past bottlenecks, and (d) connected these populations to range‐wide phylogeographic patterns.

## 2 MATERIAL AND METHODS

### 2.1 Sampling and DNA extraction

Between 2006 and 2010, core areas of the brown bear distribution in Greece were sampled using noninvasive methods. Samples were collected from the three main geographical populations of Peristeri, Pindos, and Rhodope Mountains (Figure 1). Hair was collected from wooden posts, rub trees, and other surfaces while stool samples were collected by patrolling the forest road network, by hiking trails, and by visiting abandoned orchards and pasture fields near forest margins. Blood and hair samples were also obtained from bears which were trapped for tagging (collaborating projects), as well as from found carcasses. The relevant regional forestry service was informed about all the bear mortality cases. In addition, a hair sample was made available from a dead individual recovered in the area of Mount Olympus (Figure 1), where there has been evidence of recolonization over the last 15 years.

Hair samples were placed in paper envelopes containing silica gel desiccant and stored at room temperature. Stool and tissue samples were placed in 99% ethanol, and blood plasma was kept frozen. All types of samples apart from hair were subsequently stored at −20°C until extraction. DNA was extracted from collected hair (*n* = 282), stool (*n* = 90), and blood (*n* = 10) samples with the latter used to create reference genotypes for allelic acceptance. For the stools, we followed the modified protocol of Skrbinšek et al. (2010) extending the Proteinase K (QIAGEN) incubation step at 56°C to last overnight. Eluted DNA was 200 μL for blood and 30 μL ‐ 100 μL for noninvasive material, and all samples were kept stored at −20°C until further processing.

### 2.2 Genotyping, microsatellite assessment, and composite profiling

DNA was amplified for 11 commonly used microsatellite loci G1D, G10C, G10H, G10L, G10P, G10X, Mu09, Mu10, Mu15, Mu23, Mu50, Mu59, and the SRY marker (Bellemain & Taberlet, 2004; Paetkau et al., 1998; Taberlet et al., 1996) in a single‐multiplex PCR designed specifically for individual identification and sexing of brown bears from stool samples (Skrbinšek et al., 2010). The multiplex was selected with the aim of producing a comparable dataset across the Dinaric–Pindos distribution. To ensure genotype reliability, we followed the multitube approach (Taberlet et al., 1996), repeating experiments up to eight times for each ambiguous genotype using the same conditions and primer concentrations as Skrbinšek et al. (2010) but reducing PCR volume to 2 μL. Genotyping took place in an MJ Research PTC‐225 Thermal Cycler and consisted of 1 μL of QIAGEN Multiplex PCR Master Mix (QIAGEN) and 1 μL of primer mix (forward and reverse primers combined, concentrations in Table S1) and double‐distilled H_2_O to obtain the appropriate primer concentration in the final solution. DNA was left to dry with 1 μL and 3 μL template being used for blood and noninvasive samples, respectively. For each sample, a mixture of 1.5 μL of the diluted PCR product was loaded on a plate with 8.75 μL of formamide (Applied Biosystems) and 1μL of GS500LIZ size standard (Applied Biosystems) for fragment analysis in an ABI 3,730 (Applied Biosystems) sequencer.

Genotypes were screened for recaptures, and we constructed consensus profiles based on three rules of allele acceptance (Appendix S2). A mismatch comparison between the consensus genotypes took place in Gimlet 1.0.1 (Valière, 2002) and subsequently in MSToolkit (Park, 2001) and in Genecap (Wilberg & Dreher, 2004). Generally, a *PID*_SIB_ (Evett & Weir, 1998) similarity threshold of ≥ 85% corresponded to two mismatches and in a few cases to three. *PID*_SIB_ was calculated in GenAlEx (Peakall & Smouse, 2006). Any final consensus genotype with missing information in more than three loci was deemed as unreliable and was excluded from all further downstream analyses. Our acceptance rule was selected by comparing error rates (Broquet & Petit, 2004) between datasets with a Kruskal–Wallis test (Kruskal & Wallis, 1952). Exact tests for deviation from Hardy–Weinberg equilibrium (HWE) and for linkage disequilibrium were calculated in Genepop v1.2 (Raymond & Rousset, 1995) initially using the reference genotypes and later with the whole dataset treating each genetic population separately. The frequency of null alleles for each locus in each population was estimated in FreeNa setting 0.1 as the acceptable threshold (Chapuis & Estoup, 2007). Locus G10H exhibited a high allelic dropout rate (≈ 14%), as seen by Skrbinšek et al. (2010), and so, it was removed from further analyses.

### 2.3 Analysis of population structure and isolation‐by‐distance

Two Bayesian methods were used to detect population structure: the clustering approach of STRUCTURE (Pritchard et al., 2000) and GENELAND (Guillot et al., 2005). Bayesian inference in STRUCTURE was based on 10^6^ Markov chain Monte Carlo (MCMC) simulations after a 2 x 10^5^ burn‐in period. *K* was allowed to vary from 1 to 10, and a total of 10 independent simulations were run for each *K*. We selected the admixture model with correlated allele frequencies without prior identification of subpopulations and allowed alpha to vary. Log‐likelihood [*Ln(K)*] values were plotted in Structure Harvester v0.563 (Earl & vonHoldt, 2011). Membership coefficients were grouped using the Greedy algorithm in CLUMPP (Jakobsson & Rosenberg, 2007) and visualized in DISTRUCT v 1.1 (Rosenberg, 2004). To determine the effect of relatives on the inference of genetic structure (Rodríguez‐Ramilo & Wang, 2012), we carried out a test with 56 unrelated individuals by removing individuals with ≥ 0.3 pairwise relatedness calculated in ML‐RELATE (Kalinowski & Taper, 2006) separately for each of the three putative populations. No such effect was observed so we proceeded in producing cluster membership for all unique profiles (*n* = 150). In GENELAND, a Poisson–Voronoi tessellation was run in a two‐step mode, performing a number of tests to determine the best fit of the parameters. For the first step, 10^5^ MCMC iterations were performed in five independent runs where *K* was set to vary 1–10 with thinning of 100. We used the uncorrelated model which performs better (Guillot et al., 2005) and followed default recommendations for the values of the maximum rate of Poisson process and for the maximum number of nuclei in the Poisson–Voronoi tessellation. Spatial uncertainty was set to enable the software to assign individuals in different clusters based on the average home range of an adult male brown bear which in Greece is estimated to be ≈200 km^2^ (A. Giannakopoulos unpubl. data). The modal population was selected after a burn‐in period of 10 x 100 iterations. In the second step, we selected the run with the highest posterior probability and run the analysis an additional 30 times to assign individuals into clusters, setting *K* fixed to the optimal value suggested by the first run, with all other parameters kept the same as in step one. As Bayesian inference can cause artificial clustering (Frantz et al., 2009), the dataset was tested for the presence of isolation‐by‐distance (IBD; Wright, 1943) by spatial autocorrelation analysis, equivalent to stratified Mantel tests, in SPAGeDi 1.2 (Hardy & Vekemans, 2002) using the kinship coefficient (*Fij*; Loiselle et al., 1995) as pairwise estimator of relatedness.

A jackknife procedure over loci was used to estimate standard errors. The slope was tested for a significant difference from zero by 10,000 permutations following an individual‐based approach where significance in association between genetic and geographical distance is tested by permuting the individual locations. A partial Mantel test was performed in GENODIVE (Meirmans & Van Tienderen, 2004) to test whether the higher‐level clustering would be significant after correction for IBD (Guillot et al., 2009). A model matrix of cluster membership was created where the value of “0” was allocated to a pair of individuals from the same cluster and the value of “1” to a pair of individuals from a different cluster. Association between this model matrix and the kinship coefficient was tested while correcting for geographical distance. Consensus coordinates were allocated for bears captured at multiple locations (on the basis of profile matches), excluding four individuals from the western distribution with unreliable coordinates.

### 2.4 Diversity, differentiation, and migration rates

Measures of diversity, polymorphism, and differentiation were estimated in Genodive (Meirmans & van Tienderen, 2004) for all genetic clusters, excluding the individual from Olympus as a geographical outlier. Allelic richness (*A*_R_), which is independent of sample size, was calculated in FSTAT (Goudet, 1995) and private alleles (A_P_) in GenAlEx (Peakall & Smouse, 2006). *F* _st_ reflects migration over a longer time span as it is based on heterozygosity, which is determined by common and thus usually old alleles. As within‐population heterozygosity reduces *F* _st_ (Meirmans & Hedrick, 2011), we computed the standardized fixation index *F’* _st_ (Meirmans, 2006) and estimated Jost's *D* _est_ (Jost, 2008). The effective number of migrants (*Nm*) was estimated in GENEPOP and corrected for sample size (Barton & Slatkin, 1986). We also computed contemporary migration rates (Appendix S4) through a Bayesian approach in BAYESASS 1.3 (Wilson & Rannala, 2003). The method takes into account private alleles which are given they are relatively rare and expected to have emerged recently so providing an indication of migration during a more recent time scale (Yamamichi & Innan, 2012). After an initial run with the default input parameters for migration (*dM*), allele frequencies (*dA*), and inbreeding coefficient (*dF*), we adjusted the values to 0.1, 0.25, and 0.35, respectively, to achieve acceptance rates for changes to the parameters between 40% and 60% (Faubet et al., 2007). For the main analysis, we performed a total of 10 independent runs of 10^7^ MCMC iterations after a burn‐in of 10^6^ and a thinning rate of 100, using Bayesian deviance as an optimality criterion (Spiegelhalter et al., 2002) to find the run that provided the best fit (Deviance R script; Meirmans, 2014).

### 2.5 Total and effective population size

Total population size (*N* _c_) was based on two capture histories generated for each cluster (Appendix S5) to avoid inflating of recaptures due to the occurrence of hair trap clusters in some areas. A recapture was defined as the re‐occurrence of a genotype at locations distanced at a minimum of 500 m and 1 km and only when sampled during separate sampling sessions and the reported estimate is the average of the two. We used the maximum‐likelihood approach in CAPWIRE (Miller et al., 2005) with 5,000 bootstrapping replications. The software allows for multiple captures of an individual per sampling occasion and performs well by producing narrow CI even when single sweeps have taken place and when temporal heterogeneity has occurred (Miller et al., 2005). The two innate rates model (TIRM) was selected, which assumes unequal probability between individuals and so fitted well with the heterogeneity of the multiple source sampling method. To avoid overinflating, all the profiles which corresponded to dead bears (*n* = 7) were excluded from the analysis. Effective population size (*N*_e_) was computed using the linkage disequilibrium method in LDNe, which is recommended for both unlinked and linked loci and has greater power in small sample sizes (Waples & Do, 2008). Alleles with a frequency lower than 2% were excluded from the analysis and 95% CI was determined by the jackknife method.

We examined the possibility that a population had undergone a genetic bottleneck by estimating heterozygote excess for a given level of allelic richness expected from a constant population size in Bottleneck ver. 1.2.02 (Piry et al., 1999). Alleles with a frequency lower than 2% were excluded from the analysis and 95% CI were determined by the jackknife method. We performed 5,000 iterations under the infinite allele model (IAM) and the two‐phased mutation model (TPM). TPM was assumed as a mixture of 78% of the stepwise mutation model in addition to IAM, with a variance of 12 for multirepeat mutations, which are the recommended values based on empirical evidence of microsatellite mutations (Peery et al., 2012).

### 2.6 Analysis of phylogeography

Mitochondrial control region sequences were obtained from bears in all three subpopulations of Pindos (*n* = 38), Peristeri (*n* = 10), Rhodope (*n* = 9), and for the sample from Olympus (*n* = 1). Using the primer pair 1 from Keis et al. (2013), a fragment of the mitochondrial control region was PCR‐amplified in 20 μL reactions containing 0.5 µM of each primer, 1 × Advantage 2 PCR Buffer (BD Biosciences), 0.2 mM dNTP (Fermentas), 1 × Advantage 2 Polymerase Mix (BD Biosciences), and 10–50 ng of DNA. Cycling parameters were as follows: 1 min at 95°C, followed by 35 cycles of 20 s at 95°C, 30 s at 60°C and 2 min at 68°C; and concluded with 2 min at 68°C. For purification, one unit of each of shrimp alkaline phosphatase and exonuclease I (Fermentas) was added to 10 μL of PCR postreaction mix, incubated for 30 min at 37°C, and then inactivated by 15 min at 80°C. Both DNA strands were sequenced using the same primers as for primary amplification, performing cycle sequencing in 10 μL reactions using the Big Dye Terminator v.3.1 Cycle Sequencing Kit (Applied Biosystems), following the manufacturer's protocol. Initial denaturing was at 96°C for 60 s, followed by 25 cycles at 96°C for 10 s, 50°C for 15 s, and 60°C for 4 min. Sequences were resolved on the ABI 3,730 automated DNA sequencer (Applied Biosystems). Sequences were visualized, aligned, and edited using Geneious v7.1.2 and BioEdit v7.2.5 (Hall, 1999). Haplotype and nucleotide diversity indices were calculated with dnasp (Librado & Rozas, 2009) excluding the individual from Olympus as a geographical outlier. To evaluate the phylogenetic position of Greek samples, 89 homologous sequences were included from GenBank (Appendix S6). Global analysis of molecular variance (AMOVA) estimates were produced in Arlequin version 3.5 (Excoffier & Lischer, 2010) as a means of testing for a pattern of subpopulation structure across different strata (within and between subpopulations). Phylogenetic relationships between haplotypes were inferred using median‐joining network analysis (Bandelt et al., 1999) in PopART (Leigh & Bryant, 2015).

## 3 RESULTS

### 3.1 Individual identification, error rates, and microsatellite validity

Out of 216 profiles, we identified a total of 150 consensus genotypes corresponding to unique profiles: 78 males, 66 females, and six samples for which the sex remaining undetermined. For the noninvasive samples, the sex ratio was almost even for stool samples (44% male, 49% female, 7% undetermined) whereas 74% of the hair samples collected from rub surfaces belonged to males, 23% to females, and 3% were undetermined. Most individuals were sampled in the western distributions (Pindos, *n* = 99; Peristeri, *n* = 28; Olympus, *n* = 1) and the remaining bears were sampled from Rhodope (*n* = 22). Amplification success was higher for stool (76%) than for hair (66%) samples. No locus had an estimated frequency of null alleles above 0.1. *PID*_SIB_ analysis. Waits et al. (2000) showed that five loci (G1D, G10C, G10P, Mu59, and Mu50) were sufficient to distinguish between full siblings with confidence > 0.99 and 11 loci provided a confidence > 0.999 (Appendix S1). Loci G10P, Mu59, Mu09 departed from HWE, but not consistently across all three populations and were kept. *f* _is_ values ranged from 0.021 to 0.047 (Table 1), suggesting some departure from random mating. After Bonferroni correction (Holm, 1979), linkage disequilibrium was found to be significant in 6% of the pairwise comparisons across all populations, which could be due to the strong genetic structuring.

**TABLE 1:** Estimates of the mean polymorphism and genetic diversity for 149 bears in Greece based on 11 microsatellite loci for the three genetically differentiated populations of Peristeri, Pindos, and Rhodope (*N* = sample size of each genetic cluster, *A*_L_ = mean number of alleles per locus, *A*_R_ = mean allelic richness per locus, *A*_P_ = private alleles, *H*_o_ = mean observed heterozygosity, *H*_e_ = mean expected heterozygosity, *F*_IS_ = inbreeding coefficient, Weir & Cockerham 1984)

Population	*N*	A_L_	A_R_	A_P_	H_O_	H_E_	F_IS_
Peristeri	30	5.64 ± 0.85	5.39	0.55 ± 0.25	0.65 ± 0.04	0.69 ± 0.03	0.047
Pindos	97	5.27 ± 1.62	4.57	0.36 ± 0.24	0.61 ± 0.05	0.64 ± 0.06	0.042
Rhodope	22	6.09 ± 1.50	6.03	1.46 ± 0.31	0.71 ± 0.04	0.73 ± 0.03	0.021

### 3.2 Genetic structure and clustering

**FIGURE 2:**
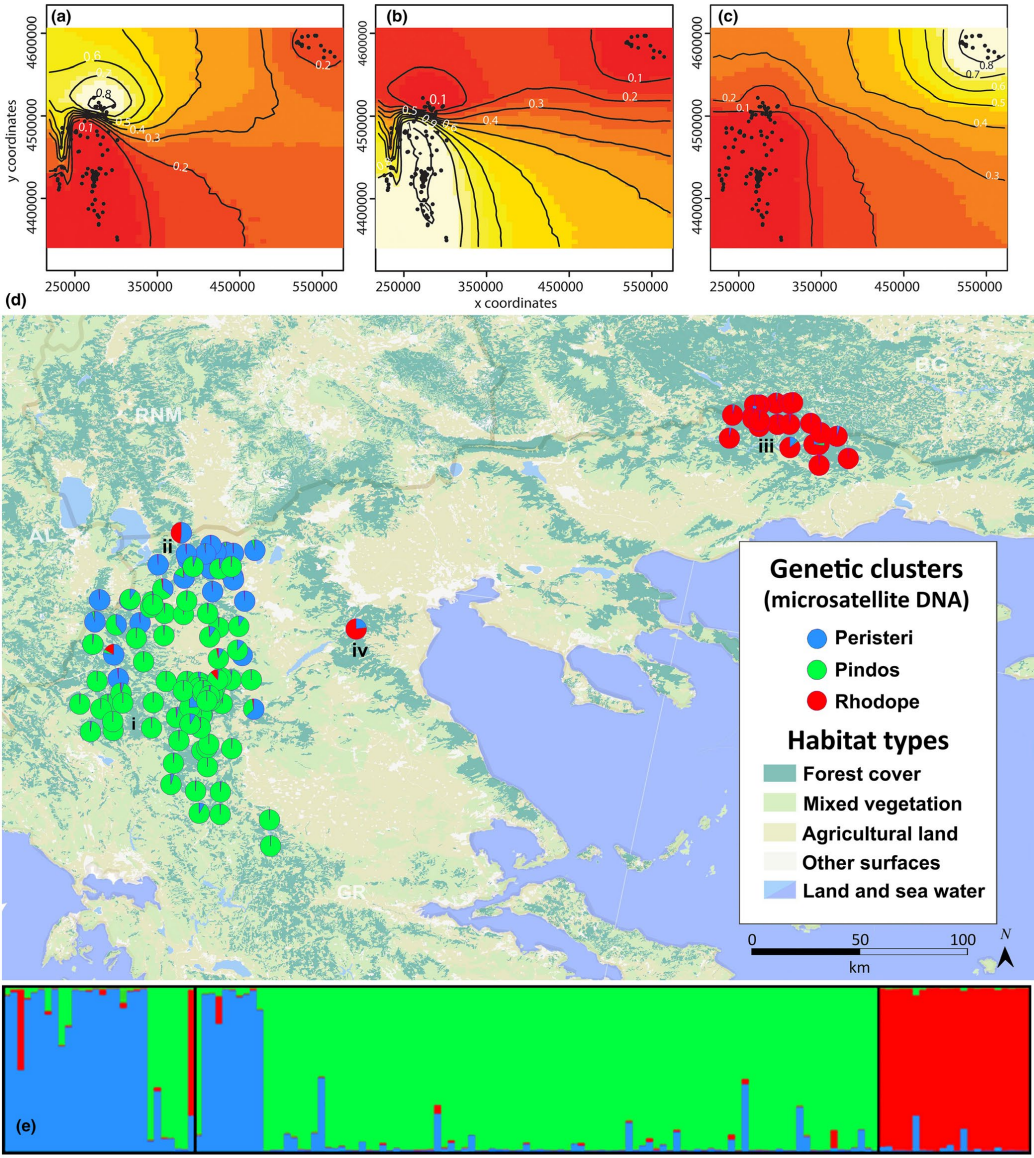
Congruent spatial population structure for the brown bears in Greece inferred by GENELAND (A, B, C) and STRUCTURE (D & E). Each point on map represents an individual brown bears (*n* = 150) from the (i) Pindos, (ii) Peristeri, (iii) Rhodope populations and one bear sampled from (iv) Olympus, with matching colors to the membership coefficient values *Q* for each cluster (E) for the best‐case scenario (*K* = 3)

In STRUCTURE, the best clustering is indicated when *L(K)* approaches the highest value but still has a low variance, increasing only slightly or starting to plateau for higher *K*. In our analyses, this occurred at *K* = 3, suggesting three subpopulations and no bias due to the presence of family groups (see Appendix S3). At *K* = 2, the *L(K)* value had larger variance than *K* = 3; however, a clear‐cut distinction of the Q values was observed between the eastern and the western population, with further substructuring resulting in genetic clusters matching the geographical distribution (Figure 2). Evanno's deltaK peak also confirmed that the optimum value of clusters was at *K* = 3 (Appendix S3) with 90% of the bears sampled in Pindos allocated in one cluster, 69% of the bears sampled in Peristeri allocated to another, and all Rhodope bears were assigned unambiguously to a third cluster. Using Q value of 0.7 as a threshold for admixture, we found five individuals with admixed ancestry, all sampled in the western distribution with a male individual sampled in Peristeri found to owe 49% of its ancestry to Rhodope bears, while another male bear that was sampled in Olympus was assigned to the Rhodope cluster (Q = 0.768).

GENELAND confirmed that the most likely number of demes was *K* = 3 (see Appendix S3), consistent with the geographical populations of Peristeri, Pindos, and Rhodope. Subsequent spatial analyses excluding the Rhodope samples verified the substructuring of the western distribution (data not shown). Samples from Rhodope, Peristeri, and Pindos were all grouped in separate clusters (see Appendix S3). Spatial autocorrelation analyses produced a weak but significant relationship between kinship and distance (*r* = −0.014, *p* < .001) for the western distribution as a whole and within Pindos (*r*= −0.006, *p* < .001) and Peristeri (*r* = −0.008, *p* = .043). The result after correction for clustering was *r* = −0.08 (*p* < .001).

### 3.3 Diversity estimates and gene flow

All loci were found to be polymorphic and the number of alleles ranged from 5 to 11 across all populations with Rhodope population being the most polymorphic in all measures (Table 1). Expected heterozygosity (*H*_E_) decreases from north to south by almost equal intervals (*H*_E_ Rhodope = 0.73 ± 0.03; *H*_E_ Peristeri = 0.69 ± 0.03; *H*_E_ Pindos = 0.64 ± 0.06). Three times as many private alleles were found in Rhodope than in the Pindos subpopulation with the relative percentage of these values being 6.9% in Pindos, 9.8% in Peristeri, and 23.9% in Rhodope. Mean *F*_IS_ across all clusters was 0.036 (*n* = 3) suggesting low inbreeding and connectivity within demes.

Pairwise *F′*_ST_ and Jost's *D*_EST_ values revealed high differentiation between subpopulations (Table 2). Bayesian estimation of migration revealed a north–south connectivity between Peristeri and Pindos bears and evidence of recent gene flow (Table 3). All ten runs converged (see Appendix S4) and the values of Bayesian deviance ranged from 8,314.4 to 8,313.04. *Nm* after correction from sample size was highest between Pindos and Peristeri (*Nm* = 1.1) and lowest between Pindos and Rhodope (*Nm* = 0.1), whereas between Peristeri and Rhodope *Nm* = 0.6.

**TABLE 2:** Pairwise *F′* _st_ (above diagonal) and Jost's *D* _est_ corrected (below diagonal) between brown bear populations of Greece

Population	Peristeri	Pindos	Rhodope
Peristeri	−	0.172	0.296
Pindos	0.127	−	0.433
Rhodope	0.240	0.348	−

**TABLE 3:** Mean (and 95% CI) recent migration rates per generation inferred by BayesAss, for the brown bear populations of Greece. The rate is the proportion of individuals that immigrated from a source population to another. Diagonal values represent nonimmigrants

From/To	Peristeri	Pindos	Rhodope
Peristeri	0.818 (0.702–0.934)	0.163 (0.05–0.275)	0.019 (0–0.051)
Pindos	0.005 (0–0.013)	0.989 (0.978–1)	0.006 (0–0.015)
Rhodope	0.021 (0–0.059)	0.015 (0–0.043)	0.964 (0.919–1)

### 3.4 Total, effective population size, and bottleneck

The total number of capture locations was 254 with the majority being singletons (Figure S5.1). Singletons may inflate population estimates (Miller et al., 2005) when clustering around the edges of a distribution; however, we did not observe such clumps in our data. The maximum distance between a recapture pair was 30 km. The total average population size was 499 (95% CI: 285–809) individuals with 299 (95%: 193–351) estimated for Pindos, 109 (95% CI: 52–196) for Peristeri and 91 (95% CI: 41–262) for Rhodope (Table 4). *N*_E_ was highest for Pindos with 97.4 (95% CI: 64.3–163.8), followed by Peristeri with 59.1 (95% CI: 32.8–181.1), and 42.2 (95% CI: 25.3–97.7) for Rhodope. All three populations exhibited signatures of recent bottlenecks under IAM and Pindos under TPM (Table 4).

**TABLE 4:** Average estimate of population size (*N*_C_), effective population size (*N*_e_), and the statistical significance of the bottleneck under the infinite alleles model (IAM) and the two‐phase mutation model (TPM) for the genetic clusters that comprise the brown bear population of Greece (*p < .01, **p < .05)

Population	*N*_C_ (95% CI)	*N*_E_	IAM	TPM
Peristeri	109 (52–196)	59.1 (32.8–181.1)	0.0337^*^	0.2598
Pindos	299 (193–351)	97.4 (64.3–163.8)	0.0046^*^	0.0269^**^
Rhodope	91 (41–262)	42.2 (25.3–97.7)	0.0007^*^	0.1392

### 3.5 Matrilineal phylogeography and mtDNA diversity

**FIGURE 3:**
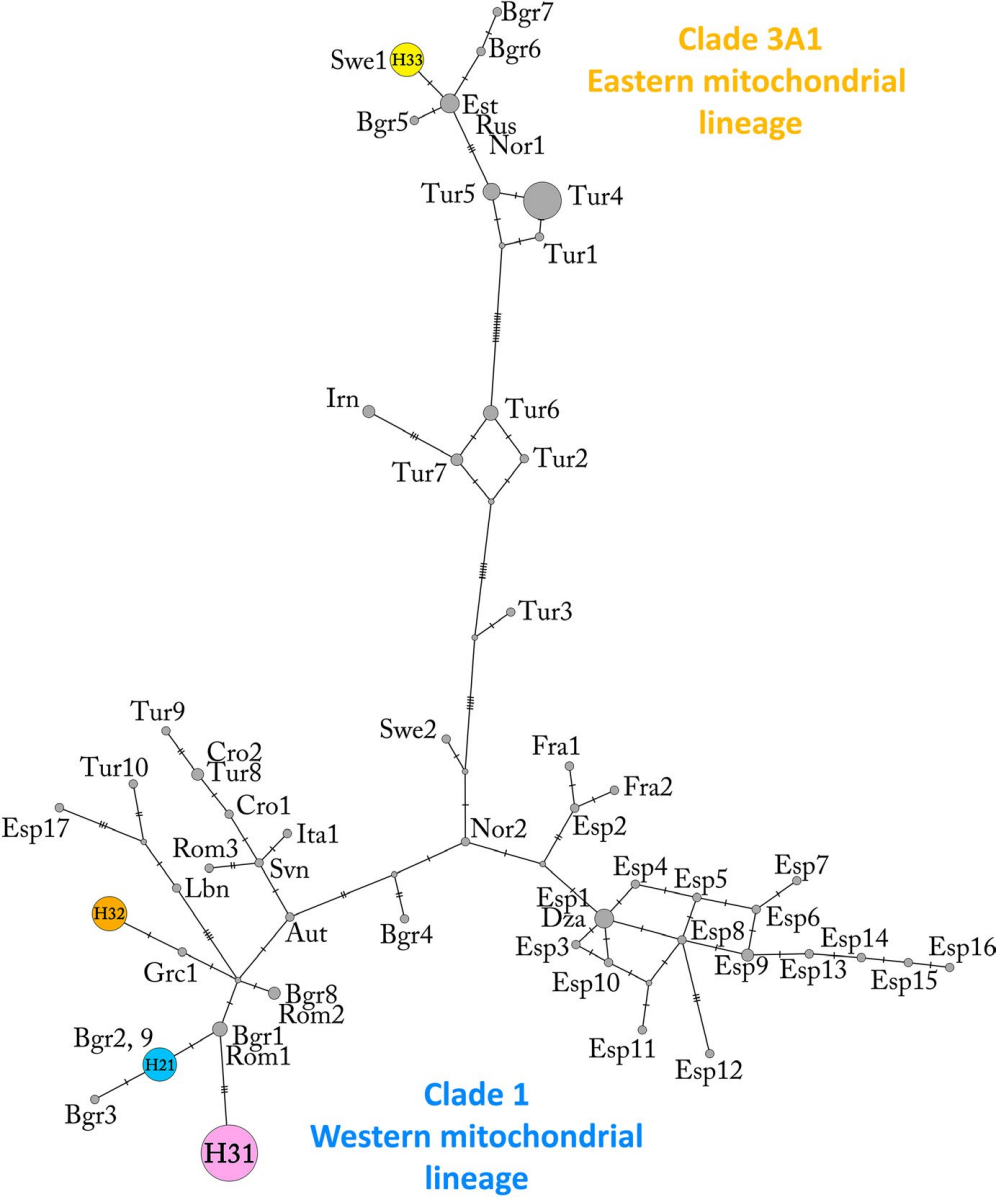
Median‐joining network of all brown bear haplotypes (mtDNA control region, 244bp; *n* = 51). Node size is proportional to the frequency of the haplotype. Colored circles represent haplotypes to which the newly sequenced samples belonged to and gray circles represent the homologous sequences from GenBank, which were used for the inference of phylogeographic relationships. Hatch marks on the lines represent the number of mutations between samples. Aut = Austria, Bgr = Bulgaria, Cro = Croatia, Dza = Algeria, Esp = Spain, Est = Estonia, Fra = France, Grc = Greece, Ita = Italy, Irn = Iran, Lbn = Lebanon, Nor = Norway, Rom = Romania, Rus = Russia, Svn = Slovenia, Swe = Sweden, Tur = Turkey. The colors of the symbols correspond to the ones used in the haplotype distribution map (Figure 5)

We obtained mitochondrial control region sequences for 57 samples: Pindos (*n* = 38), Peristeri (*n* = 9), Rhodope (*n* = 9), and Olympus (*n* = 1). Trimming and alignment with homologous sequences from GenBank yielded a dataset of 51 haplotypes with a final sequence length of 244 bp, spanning pos. 15452–15716 according to reference sequence HQ685901 (pos. 15526–15542 containing the pyrimidine tract were excluded due to high level of homoplasy). The analyzed samples yielded four haplotypes in total (no 21, 31, 32, and 33 in Figure 3), with 21 described before in Greece (Pylidis, 2015), while haplotype 31 and 32 were globally new (GenBank accession numbers KR021974–KR021975). Haplotype 33 had been detected in bears from Sweden (HE657212; Hailer et al., 2012) and haplotype 21 in Bulgaria (AP012591; Hirata et al., 2013; KJ638592; Frosch et al., 2014). Haplotypes 21, 31, and 32, detected in 95% of the analyzed samples, belong to the Western mtDNA lineage (clade 1). Haplotype 21 (*n* = 9) was found in bears exclusively from Rhodope; haplotype 31 (*n* = 38) was carried by 36 bears from Pindos and three from Peristeri, whereas haplotype 32 (*n* = 7) was confined to Peristeri and the single bear from Olympus (Figure 5). Three bears, two from Pindos and one from Peristeri, were found to carry haplotype 33 (*n* = 3), which belongs to the Eastern mtDNA lineage (clade 3a1) (Anijalg et al., 2018; Davison et al., 2011; Hirata et al., 2013). Haplotype diversity was 0.502 ± 0.07 (*H*_D_ ± *SD*) while nucleotide diversity was 0.0133 ± 0.0028 (*π* ± *SD*) for the whole Greek population (Table 5). AMOVA analysis showed that most of the diversity in the sample lies between geographical population ranges though considerable variation lies within demes as well (Table 6).

**TABLE 5:** Diversity estimates for 244‐bp fragment of the mtDNA control region for 57 brown bears from the genetic clusters that comprise the brown bear population of Greece

Population	*n*	Haplotypes	Haplotype diversity ± *SD*	Nucleotide diversity ± *SD*
Pindos	38	2	0.102 ± 0.065	0.006 ± 0.004
Peristeri	10	3	0.6 ± 0.131	0.021 ± 0.007
Rhodope	9	1	0.000 ± 0.000	0.000 ± 0.000

**TABLE 6:** Analysis of molecular variance (AMOVA) for 57 brown bears from Greece

Source of variation	*df*	Sum of squares	Variance components	% Variation
Among populations	2	43.740	1.404 (VA)	60.54
Within populations	55	50.708	0.922 (VB)	39.46
Total	57	94.448	2.336	

## 4 DISCUSSION

### 4.1 Detecting biologically meaningful population structure

In the first comprehensive multi‐source noninvasive genetic study at range scale of the brown bear population in Greece, utilizing both microsatellite and mtDNA markers we report the presence of three highly differentiated genetic groups (Figure 2). Using two methods of Bayesian clustering, we determined that the geographical populations of Peristeri, Pindos, and Rhodope host distinct genetic demes. According to STRUCTURE analysis, a clear‐cut distinction between the eastern and western distribution was observed and a further partitioning of the western distribution into two demes with Ln *(K)* and deltaK agreeing on the optimum number of clusters (*K* = 3).

The genetic clusters match the geographical populations, with some overlapping in the northwest of the Pindos mountain range in the Mt. Grammos (N40.35° E20.85°) where the habitat creates corridors for dispersing bears to use. GENELAND supported the case for *K* = 3 illustrating in more detail the spatial pattern of the structuring and the contact zones of the populations inferred by the microsatellites (Figure 2; panels ABC). GENELAND performed well in the presence of IBD which has been shown to create false positives leading to an incorrect identification of genetic clusters (Perez et al., 2018) especially when uneven sampling takes place (Puechmaille, 2016). This artifact, however, occurs in much higher levels than reported here when *r* = −0.02 (Frantz et al., 2009) which indicates that the genetic partitioning occurs over and above IBD. When it comes to population management, an under‐ or overestimation has serious conservation consequences and we took steps to ensure against artifacts using multiple analytical tools that are based on different assumptions. At *K* = 2, the Pindos population also remained distinct with the Rhodope and Peristeri bears being appointed in the same cluster (Pylidis, 2015).

### 4.2 Revealing the connectivity of the Pindos‐Dinara and East Balkan distributions

The detection of two male bears in the western distribution with Rhodopean ancestry illustrates for the first time to our knowledge, recent demographic and genetic connectivity between the Pindos and Rhodope distributions in Greece and, by extension, connectivity of the wider Dinaric‐Pindos and East Balkan populations. Bears are capable of dispersing over long distances with a proven capacity to overcome barriers such as water bodies and highways (Kaczensky et al., 2003; Paetkau et al., 1998). Elsewhere long‐range displacement of males up to 360 km has been recorded (Bartoń et al., 2019) meaning that the approximate 220 km which separates Peristeri from Rhodope is well within the dispersal capacity of the species. This result highlights the need to conserve the corridors which allow the connectivity of the bear populations over large distances in the fragmented and human‐saturated landscape of the Balkans.

While a differentiation between the bears of the eastern and western distribution was expected due to their long‐term apparent geographical fragmentation, the high level of genetic differentiation between the geographically adjacent Peristeri and Pindos was a surprise. Their pairwise value for the western demes (*Nm* = 1.1, *F’*_ST_ = 0.172) is not far from the migration fixation index which predicts that when *F*_ST_ = 0.2 then *Nm* is expected to be ≤ 1). A possible explanation for the observed differentiation is that the mountainous terrain in Northern Greece may act as a physiographic barrier limiting dispersal between Peristeri and Pindos and in conjunction with the female philopatry; further enhance differentiation. Mountain ranges act as biogeographical barriers preventing gene flow and inducing differentiation even in highly mobile species with large home ranges and long‐distance dispersal capacity (e.g., Korsten et al., 2009; May et al., 2008; Razgour et al., 2013; Weckworth et al. 2013).

### 4.3 The level of diversity suggests gene flow exchange between genetic clusters

Rhodope bears were found to harbor the highest genetic diversity (*H*_E_ = 0.73, Table 1) with a value close to the one reported on the contiguous Bulgarian bear population (*H*_E_ = 0.74, Frosch et al., 2014). Peristeri, despite its smaller sample size being three times smaller than Pindos, displayed higher diversity values (Tables 1 and 4) which suggested connectivity with the larger Balkan distribution. Pindos has one of the lowest *H*_E_ values among European populations (see Table S7 in Appendix), but much higher than the other populations of Mediterranean Peninsulas. Perez et al. (2009) report *H*_E_ = 0.25 for the eastern subpopulation and *H*_E_ = 0.45 for the western subpopulation; and Zachos et al. (2008) report *H*_E_ = 0.46 for the Apennine bears. It is possible that the exchange of genetic material with Peristeri may have provided the buffering effect Pindos bears needed to maintain relatively high levels of diversity contrary to the small Iberian and Italian populations, both of which are geographically isolated and have suffered a profound population reduction over the last centuries (Ciucci & Boitani, 2008; Perez et al., 2009).

Out of the three clusters, Pindos bears were found to carry the lowest number of alleles, including private ones (*A*_P_). As unique alleles arise faster through mutation under conditions of limited gene flow and genetic isolation (Slatkin & Takahata, 1985), one would expect a higher number of unique alleles in the Pindos population. This indicates that genetic bottlenecks may have been more severe in the western distribution, ultimately affecting the number of alleles due to founding effects. The low *A*_R_ value in the Pindos population, despite its largest population size, supports this notion. Relatively, low *F*_IS_ values (Table 1) indicate that overall inbreeding does not seem to be a threat, and if current conditions should persist, *H*_E_ would be expected to increase over time.

### 4.4 The first distribution‐wide population estimation for the brown bears in Greece

Our demographic analyses produced a total combined population size of approximately 500 bears (Table 4). This is the first multi‐source DNA‐based estimate for the brown bear population throughout its current distribution in Greece. Out of all three clusters, the largest total and effective population is found in Pindos (Table 4). The values we report here should be considered a minimum estimate, as the areas with low apparent density and where the distribution is expanding were not sampled. The earliest contemporary estimates suggested a size of 100–220 bears in total but these were not based on DNA analysis but inferred population size from counting females with cubs‐of‐the‐year and followed overconservative models (Mertzanis, 1999). A past attempt to estimate abundance using DNA‐based methods reported a size of 200‐250 individuals (Karamanlidis, 2011) but that census was confined to sampling clumps in localized study areas of the western distribution. Furthermore, it was based on single‐source sampling (hairs) a method which favors male‐biased sex ratio, resulting in significantly lower detection rate and can produce an underestimate of up to 25% (Bellemain et al., 2005; Boulanger et al., 2008). In our study, we used a variety of sampling methods and data sources when used simultaneously increase accuracy levels (Boulanger et al., 2008; Ebert et al., 2010; Perez et al., 2014; Sawaya et al., 2012; Stetz et al., 2014). Though the average number of captures per individual was less than the recommended value (Miller et al., 2005) resulting in wide CIs, the two capture histories were consistent in their result. Since the figures reported here are well below the *N*_E_ thresholds needed to ensure the long‐term survival of a population (500–1000; Frankham et al., 2014), its protection and monitoring by regional and national agencies should continue towards meeting this threshold.

### 4.5 The Hellenic Peninsula marks a new matrilineal contact zone for European bears

**FIGURE 4:**
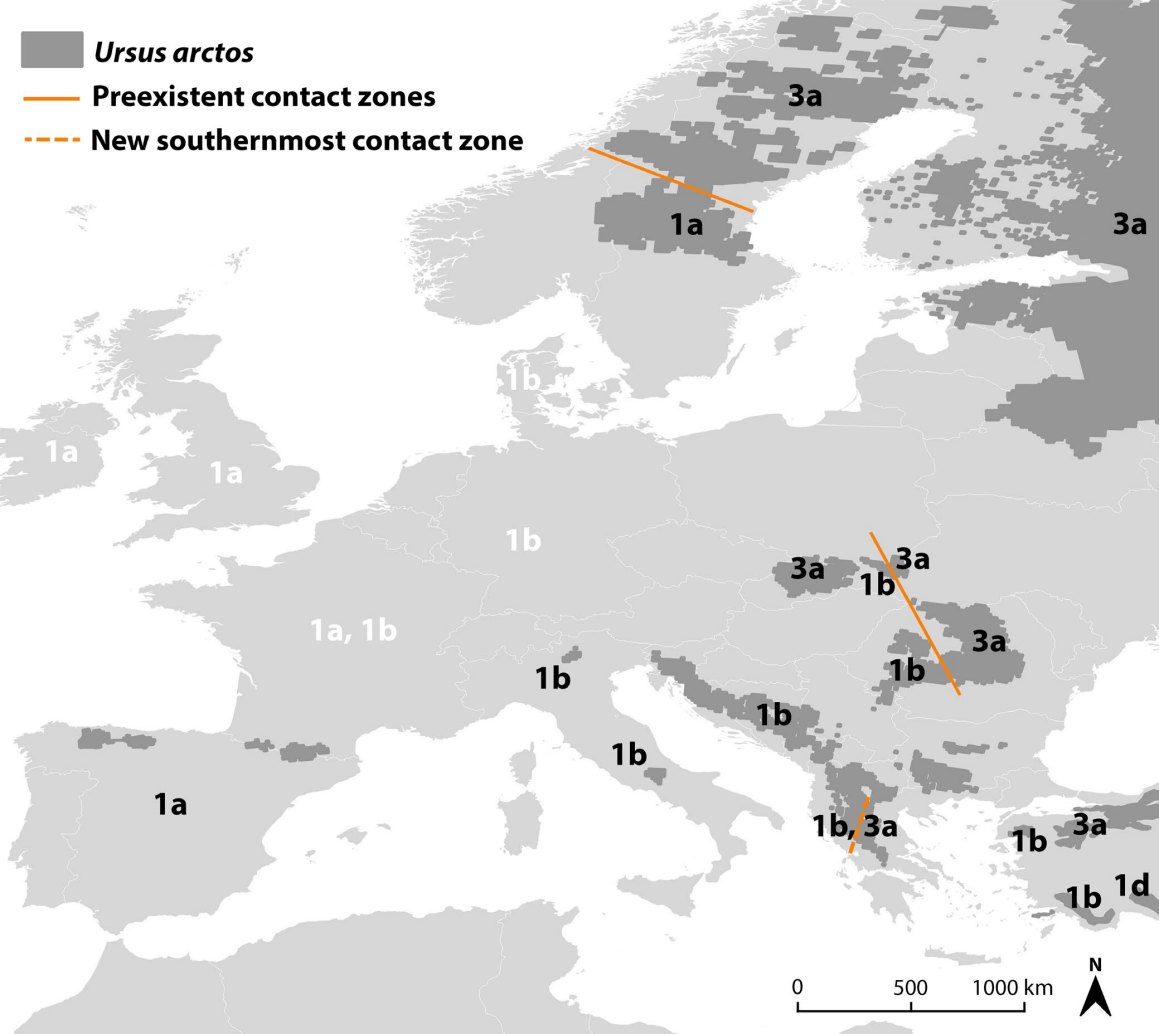
Map showing mtDNA clade contact zones association for contemporary (black text) and extinct (white text) *Ursus arctos* populations in Europe (Data taken from Davison et al., 2011; Bray et al., 2013; Çilingir et al., 2016; Matosiuk et al., 2019). Clades 1a and 1b belong to the Western mtDNA lineage and 3a1 to the Eastern mtDNA lineage. The detected clade 3a1 haplotype in Greece marks a novel contact zone for the species in Europe

In the first detailed phylogeographic study of the species throughout its current distribution in Greece, we confirm the findings of past work which placed Greek brown bears under the Western mtDNA clade 1 (Bray et al., 2013; Davison et al., 2011; Taberlet & Bouvet, 1994). The predominant findings of this work support this notion as the vast majority of the bears in Greece in our sample size were found to carry haplotypes 21, 31, 32 that belong to the Western mtDNA clade 1, with the latter two being globally new (GenBank accession numbers: KR021974–KR021975). In addition, two bears in Pindos and one in Peristeri were found to carry a fourth haplotype (H33) that belongs to clade 3a1 (Davison et al., 2011) and is closely related to the one from Scandinavia (Figure 3). This unexpected result marks the Hellenic Peninsula as the southernmost region the Eastern lineage has been detected and draws a new southernmost secondary contact zone between Eastern and Western mtDNA lineages in Europe (Figure 4). One possible explanation is that Eastern and Western mtDNA lineages have coexisted in Greece for a long time. However, as only a few bears were found to carry haplotype 33, it is more likely that the Eastern lineage has appeared due to migrations from the Carpathian refugium (Saarma et al., 2007) in relation to the LGM or from an unknown refugium in the east (Anijalg et al., 2018; Davison et al., 2011) since H33 from Greece is closely related to the haplotype from Scandinavia. An alternative scenario is that the Eastern mtDNA lineage bears originate from Turkey; however, the genetic distance between the Turkish and Greek bears is larger compared to the Greece and North European bears, therefore making this scenario less likely (Figure 3).

Apart from natural dispersal, the influence of human translocation could have played a role. Translocations of bears and other carnivores occurred in the period of the Roman empire when many thousands of large predators, including brown bears, brought to gladiator shows to fuel animal–animal and animal–human fights (Kalof, 2007). Although the main wildlife extraction route was between N. Africa and Europe, there is evidence of transporting bears along the Danube corridor as well (Spassov & Spiridonov, 1999). In recent years, bears have been captured for wealthy individuals and kept captive in their private "mini‐zoos"(Nowak et al., 2014). While in all these scenarios bear and other animals were captured for fighting in arenas or for display, it is possible that some animals carrying an Eastern lineage haplotype managed to escape and passed it on in the local gene pool.

**FIGURE 5:**
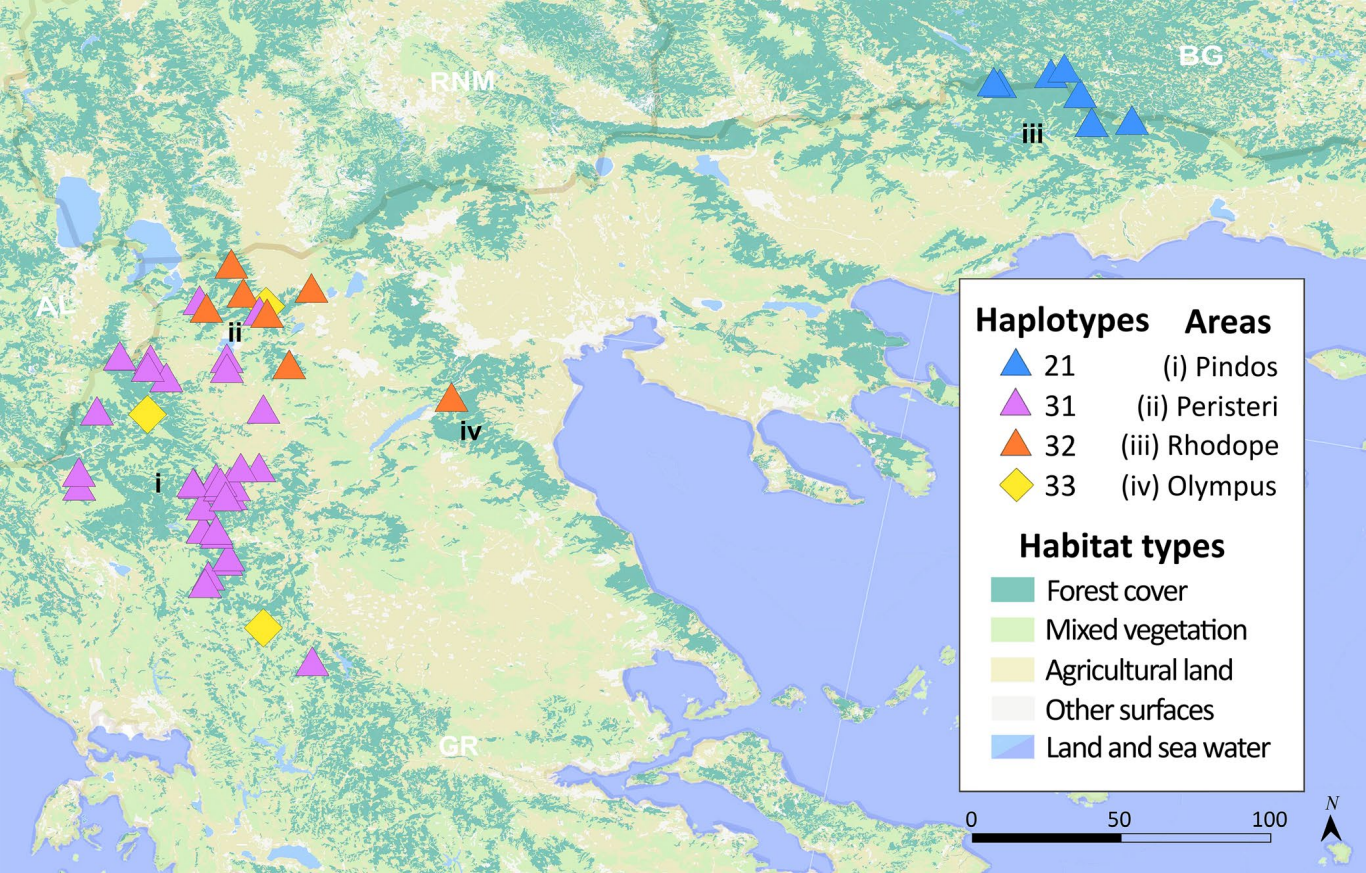
Spatial distribution of the four haplotypes detected in 57 brown bears from Greece. Triangles and diamonds are being used to visualize haplotypes belonging to clade 1 and clade 3a1, respectively, and the colors of the symbols correspond to the ones used in the median‐joining network tree in Figure 3

The results of the mtDNA analysis indicate some degree of congruence with the microsatellite data (Figures 2 and 5). AMOVA tests showed that most of the diversity lies between geographical populations/mountain ranges, though considerable variation lies also within populations (Table 6) and spatial fixation of the predominant haplotypes of the Western mtDNA lineage with the mountain ranges was strong (H31 was found in 92% of Pindos bears, H32 in 85% of Peristeri bears, and H21 in all Rhodope bears). Based on the notion that mtDNA patterns indicate ancient phylogeographical processes which possibly originated after the last glaciations (Bray et al., 2013; Davison et al., 2011), two postulations can be inferred. Firstly, that the natural factors which were suggested as the cause for the genetic structuring of the Greek brown bear population, such as limited gene flow due to geophysical barriers and female philopatry, may have been operating since ancient times. It should be noted that contemporary structuring could underlie historical patterns even in the case of extreme human‐induced bottleneck such as in the Scandinavian bear population (Xenikoudakis et al., 2015). Secondly, the population may have maintained some of its ancient diversity due to its relative isolation that may have resulted in a genetically distinct Pindos subpopulation.

## 5 CONCLUSIONS

In this study, we utilized mutlisource noninvasive sampling coupled with nuclear and mtDNA to provide a comprehensive analysis Europe's southern fringe brown bear population contributing new insights to the genetic and population history of the bears Greece. We portrayed both contemporary and historical gene flow dynamics of a highly structured population and uncovered the previously unknown connectivity of the Balkan distribution. We estimated the population size for the entirety of its distribution in Greece and described its phylogeography in detail for the first time. We report new endemic haplotypes and discovered the presence of a haplotype that belongs to the Eastern mtDNA lineage, thus revealing a new southernmost matrilineal contact zone in the Hellenic Peninsula. Congruence between nuclear and mtDNA data suggests that ancient biogeographical processes are still possibly at play in shaping the genetics of bears in the Hellenic peninsula. The observed population structuring and distinct allelic frequency of the Pindos population create interesting questions for a more detailed investigation focusing on the nature of its genetic diversity. Interestingly, high intraspecific genetic differentiation among neighboring populations is expected in marginal populations (Eckert et al., 2008) and in rear‐edge species located in former glacial refugia whenever mountainous regions are present at low latitudes (Hewitt, 2000; Petit et al., 2003). In these conditions of demographic stability, genetic variation is expected to have become geographically structured among distinct mountainous ranges (Schmitt, 2007), a pattern observed here by both the microsatellite and mitochondrial data. The relative ecological stability and habitat heterogeneity seen in Mediterranean Peninsulas stimulates and maintains genetic differentiation among populations, making them biodiversity hotspots with high levels of endemism (Blondel & Aronson, 1999; Griffiths et al., 2004; Hampe & Petit, 2005; Tzedakis et al., 2002). The occurrence of endemic haplotypes, their association with mountainous ranges (H31 with Pindos and H32 with Peristeri), and the high genetic differentiation between the two brown bear western subpopulations are consistent with those notions.

Rear‐edge populations are vital long‐term stores of distinct adaptive genetic diversity (Hampe & Petit, 2005; Petit et al., 2003). The ecologically stable areas in the Pindos Mountains for example have played a decisive role in providing refuge for numerous plant and animal species (Tzedakis et al., 2002). Identifying sources of adaptive diversity seems important particularly in the event of rapid climate change. Though larger‐bodied species with long dispersal ability are on average more likely to shift their distributions in response to the changing climate (Lyons et al., 2010), the heterogeneous landscape of the Balkans could pose a significant barrier to dispersal. Preserving genetic diversity and facilitating population connectivity and gene flow are one of the major conservation challenges for the small but persistent population of brown bears in the low‐latitude margin of their European range.

## CONFLICT OF INTEREST

None declared.

## AUTHOR CONTRIBUTIONS

**Charilaos Pylidis:** Conceptualization (lead); Data curation (lead); Formal analysis (lead); Funding acquisition (lead); Investigation (lead); Methodology (lead); Resources (lead); Validation (lead); Visualization (lead); Writing‐original draft (lead); Writing‐review & editing (lead). **Peeter Anijalg:** Formal analysis (equal); Investigation (equal); Validation (equal); Writing‐original draft (equal); Writing‐review & editing (equal). **Urmas Saarma:** Formal analysis (equal); Investigation (equal); Validation (equal); Writing‐original draft (equal); Writing‐review & editing (equal). **Deborah A. Dawson:** Methodology (equal); Supervision (equal); Validation (equal). **Nikoleta Karaiskou:** Methodology (supporting). **Roger Butlin:** Methodology (supporting); Supervision (supporting). **Yorgos Mertzanis:** Project administration (supporting); Resources (supporting). **Alexios Giannakopoulos:** Investigation (supporting); Resources (supporting). **Yorgos Iliopoulos:** Investigation (supporting); Resources (supporting). **Andrew Krupa:** Data curation (supporting); Methodology (supporting); Validation (supporting). **Terence A. Burke:** Resources (supporting); Supervision (supporting).

## Supporting information

Appendix S1–S7

## Data Availability

mtDNA sequences: GenBank accession numbers KR021974–KR021975. Microsatellite genotypes are available on DRYAD: https://doi.org/10.5061/dryad.cvdncjt3m
