# Supplementary material for: Multisource noninvasive genetics of brown bears (Ursus arctos) in Greece reveals a highly structured population and a new matrilineal contact zone in southern Europe
Source: Ecol Evol. 2021 May 2;11(11):6427–43. doi: 10.1002/ece3.7493 (PMC8207399; doi:10.1002/ece3.7493)
Supplement: Supplementary file 1 — Appendix S1–S7 [file ECE3-11-6427-s001.docx]

**Appendix S1.** Microsatellite amplification

**Table S1.** Locus names, primer sequences, dyes (6FAM, NED, PET, VIC), PCR primer concentrations and fragment sizes used in one single single-step multiplex for the genotyping of brown bear DNA.

|  |  |  |  | **Allele size range (bp)** | |
| --- | --- | --- | --- | --- | --- |
| **Locus** | **Forward primer** | **Reverse primer** | **C [μM]** | **Slovenia^S^** | **Greece^G^** |
| Mu50^B^ | GTCTCTGTCATTTCCCCATC | 6FAM-AACCTGGAACAAAAATTAACAC | 0.06 | 79–103 | 80–106 |
| Mu10 ^B^ | ATTCAGATTTCATCAGTTTGACA | 6FAM-TCAGCATAGTTACACAAATCTCC | 0.19 | 114–130 | 113–131 |
| Mu59 ^B^ | GCTCCTTTGGGACATTGTAA | NED-TGACTGTCACCAGCAGGAG | 0.15 | 97–121 | 92–120 |
| Mu23 ^B^ | NED-TAGACCACCAAGGCATCAG | GCCTGTGTGCTATTTTATCC | 0.07 | 142–156 | 142–155 |
| SRY ^B^ | GAACGCATTCTTGGTGTGGTC | PET-TGATCTCTGAGTTTTGCATTTG | 0.06 | 75 | 81 |
| G10L ^B^ | PET-ACTGATTTTATTCACATTTCCC | GATACAGAAACCTACCCATGCG | 0.1 | 156–166 | 144–164 |
| G1D ^P^ | ATCTGTGGGTTTATAGGTTACA | 6FAM-CTACTCTTCCTACTCTTTAAGAG | 0.25 | 168–182 | 166–180 |
| G10H ^P^ | 6FAM-CAACAAGAAGACCACTGTAA | AGAGACCACCAAGTAGGATA | 0.2 | 221–257 | 221–255 |
| G10C ^P^ | VIC-AAAGCAGAAGGCCTTGATTTCCTG | GGGACATAAACACCGAGACAGC | 0.05 | 97–116 | 92–113 |
| G10X^TP^ | 6FAM-CCCTGGTAACCACAAATCTCT | TCAGTTATCTGTGAAATCAAAA | 0.4 | 132–154 | 131–156 |
| Mu15 ^T^ | PET-CTGAATTATGCAATTAAACAGC | AAATAAGGGAGGCTTGGGT | 0.15 | 117–131 | 120–133 |
| G10P ^T^ | TACATAGGAGGAAGAAAGATGG | VIC-AAAAGGCCTAAGCTACATCG | 0.09 | 122–150 | 127–145 |
| Mu09 ^T^ | AGCCACTTTGTAAGGAGTAGT | VIC-ATATAGCAGCATATTTTTGGCT | 0.07 | 174–206 | 188–203 |

^T^ Taberlet *et al.* (1997) ^P^ Paetkau *et al.* (1998) ^B^ Bellemain and Taberlet (2004) **^S^** Skrbinšek *et al.* (2010) **^G^**This study, C, primer concentration (F & R) as used in PCR (see results section)


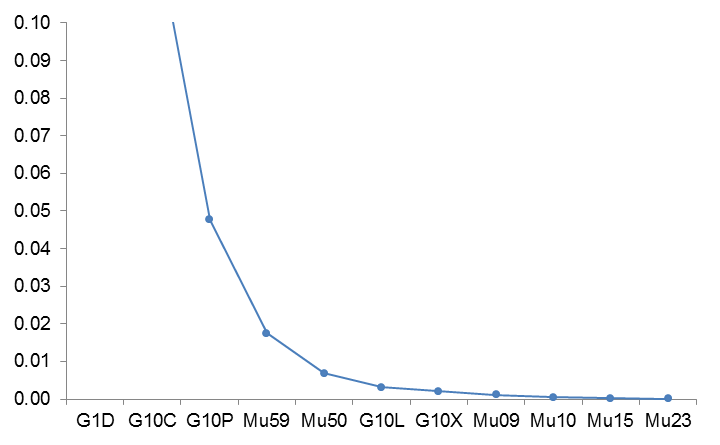


**Figure S1.** Sibling probabilities of identity (*PID_SIB_*) for 11 microsatellite loci based on brown bear (*Ursus arctos*) individuals (*n* = 150) from Greece

**Appendix S2.** Consensus genotypes

One of the goals of this study was to produce a dataset with “true” genotypes. Incorrect allele frequencies may result in false estimates of inbreeding, migration rates and false detection of population bottlenecks (Pompanon *et al.* 2005), while measures of polymorphism are expected to vary depending on the number of alleles. Therefore, efforts should be taken to minimize any bias in allelic frequencies created by the inclusion or rejection of alleles and to produce profiles as close as possible to the theoretical true genotype (Paetkau 2003; Bonin *et al.* 2004; Pompanon *et al.* 2005). The attempt to minimize genotyping error and deal with poor quality samples was achieved by a applying a rigorous data screening workflow in which the dataset was inspected for scoring errors (Fig. S2).

Consensus genotypes were constructed manually for each sample from each repeat PCR for all types of sample. In this study, to determine if there was cause to relax the allele inclusion criteria consensus profiles were constructed using three rules, followed by a statistical comparison of the error rates between the respective datasets. For the first rule homozygotes and heterozygotes alleles need appear twice to be accepted in a similar manner to Adams and Waits (2007) who showed that relaxing the criteria for both homozygous and heterozygous results does not undermine data quality by producing false genotypes. For the second rule singleton allele of a non-invasive genotype was accepted if it matched an allele of a reference genotype and/or a non-invasive genotype of a non-invasive sample of an independent PCR. The second rule was a variation of the Skrbinšek *et al.* (2010). A final dataset was constructed by accepting all alleles. Unique alleles and single allele jumps were identified using Excel Microsatellite Toolkit 3.3.1 (Park 2008) and were assessed by cross-referencing with the raw data in GeneMapper and the minimum peak threshold for each allele was 100 relative fluorescent units (RFU). Checks were performed for the whole dataset as well as for the two geographical groups until there was no suspicion left that the remaining alleles were misidentified.

Raw data

Peak calling (>100 RFU)

Export genotypes table

Check database of consensus genotypes for all rules, looking for one base pair jumps and unique alleles using appropriate software eg. Microsatellite Toolkit 3.3.1

eg. Adams & Waits 2007

Create consensus profiles by pooling genotypes from replicate PCRs of the same sample using an appropriate rule for accepting an allele

eg. Skrbinšek *et al.* 2010

eg. Frantz *et al.* 2003

Does in any data set a unique/uncommon allele appear in two independent PCRs? Cross reference with raw data

**No**

Return to raw data and delete peak calling

**Yes**

Accept allele. If biologically meaningful split each dataset into geographical units and repeat process.

Remaining unique or uncommon alleles could be location specific

Produce error rates using an appropriate method (eg. Broquet & Petit 2004) and proceed in statistical comparison between all rules

Choose the appropriate data set based on the results of the statistical comparison

Compare consensus profiles for up to 3 mismatches using multiple software eg. GIMLET, Microsatellite Toolkit 3.3.1

**Consensus profiles**

**Fig S2** A flow chart of the genotype data filtering method used in this study.

**Table S2** Comparison of allelic dropout (ADO) and false alleles (FA) between the three rules that were used to accept an allele as part of the sample set selection decision making process

| Type Error | Rule_1_ | Rule_2*_ | Rule_3*_ | Significance |
| --- | --- | --- | --- | --- |
| ADO | 0.067 | 0.069 | 0.071 | χ^2^=0.004, 2 d.f., p = 0.998 |
| FA | 0.029 | 0.025 | 0.017 | χ ^2^=1.692, 2 d.f., p = 0.429 |
| N | 224 | 247 | 254 |  |
| ^*^Adams & Waits (2007) ^*^Skrbinšek *et al.* (2010) ^*^This study | | | | |

**References**

Adams JR, Waits LP (2007). An efficient method for screening faecal DNA genotypes and detecting new individuals and hybrids in the red wolf (*Canis rufus*) experimental population area. Conservation Genetics, **8**, 123–131.

Bonin A, Bellemain E, Eidesen PB *et al.* (2004) How to track and assess genotyping errors in population genetics studies. *Molecular Ecology*, **13**, 3261–3273.

Frantz AC, Pope LC, Carpenter PJ, Roper TJ, Wilson GJ, Delahay RJ, Burke T (2003) Reliable microsatellite genotyping of the Eurasian badger (*Meles meles*) using faecal DNA. *Molecular Ecology*, **12**, 1649–1661.

Kenta T, Gratten J, Haigh NS, Hinten GN, Slate J, Butlin RK, Burke T (2008) Multiplex SNP-SCALE: a cost-effective medium throughput single nucleotide polymorphism genotyping method. *Molecular Ecology Resoures*, **8**, 1230–1238.

Paetkau D (2003) An empirical exploration of data quality in DNA-based population inventories. *Molecular Ecology*, **12**, 1375 –1387.

Pompanon F, Bonin A, Bellemain E, Taberlet P (2005) Genotyping errors: Causes, consequences and solutions. *Nature Reviews Genetics*, **6**, 847–859.

Kruskal WH and WallisWA (1952) Use of ranks in one criterion variance analysis. *J.Amer.Statist.Ass.*, **47**, 583–621.

Skrbinšek T, Jelenčič M, Waits L, Kos I, Trontelj P (2010) Highly efficient multiplex PCR of noninvasive DNA does not require pre-amplification. *Molecular Ecology Resources*, **10**, 495–501.

Taberlet P, Griffin S, Goossens B *et al*. (1996). Reliable genotyping of samples with very low DNA quantities using PCR. *Nucleic Acids Research*, 24, 3189–3194.

**Appendix S3.** Population structure


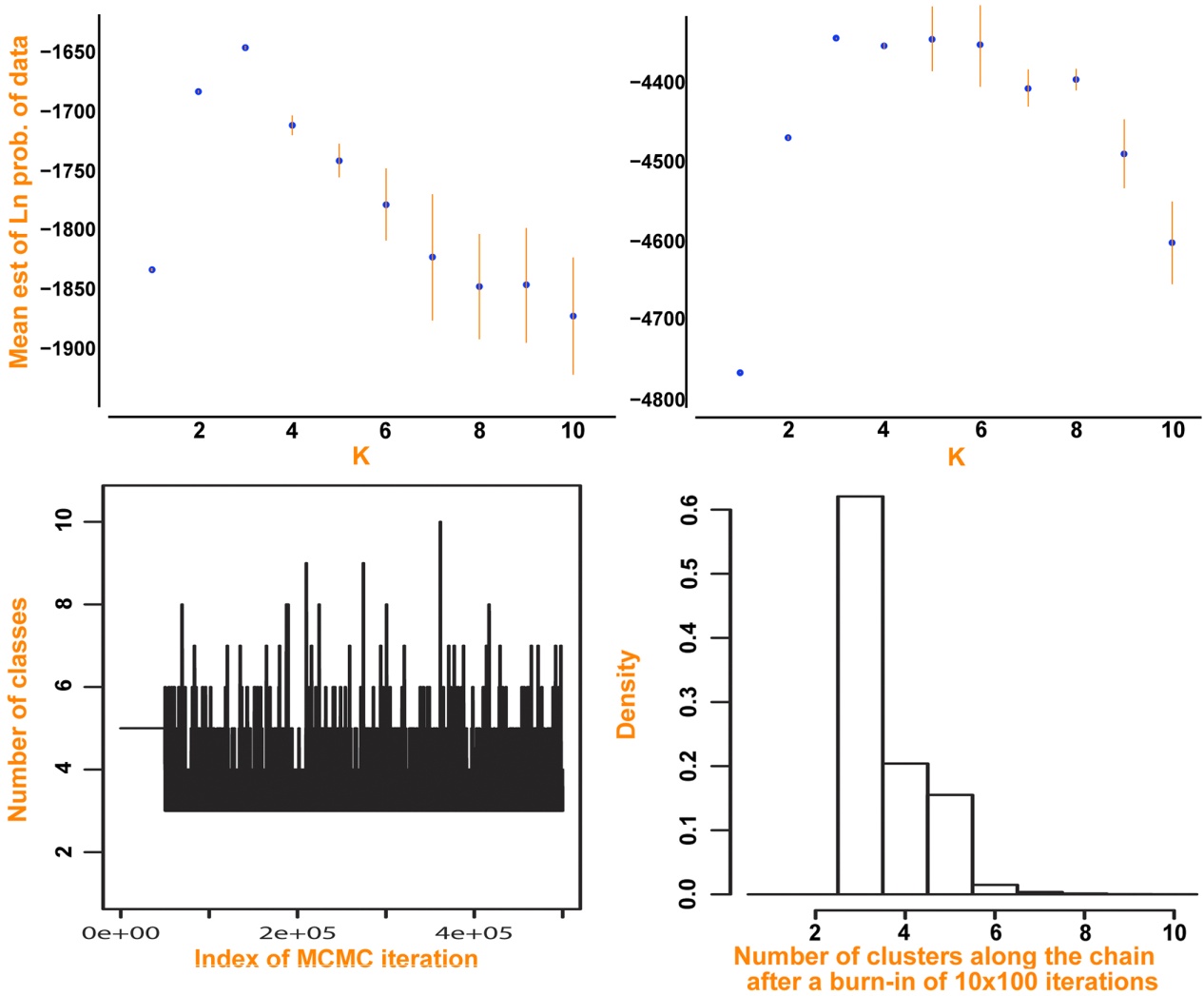


**Figure S3.1** Number of clusters indicated by STRUCTURE (top) and GENELAND (bottom). Mean L (K) (±SD) over 10 runs for each *K* value for the whole data set (top right) and for the subset of the data without relatives (top left). Both Bayesian methods indicated the best clustering occurred at *K* = 3.


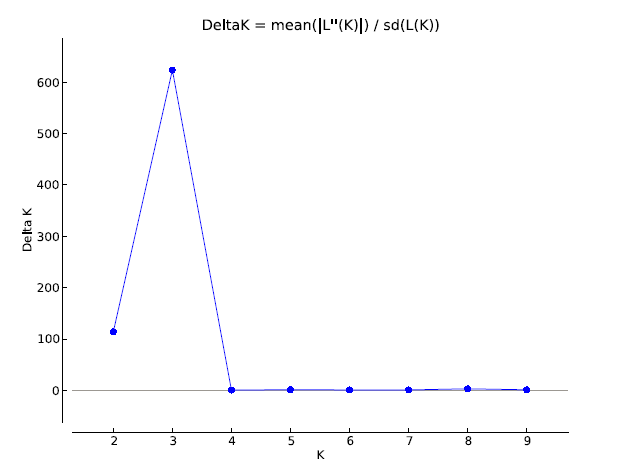


**Figure S3.2** Evanno’s Delta K showing K = 3 as the most optimum clustering scenario generated by STRUCTURE

**Appendix S4.** Migration rates


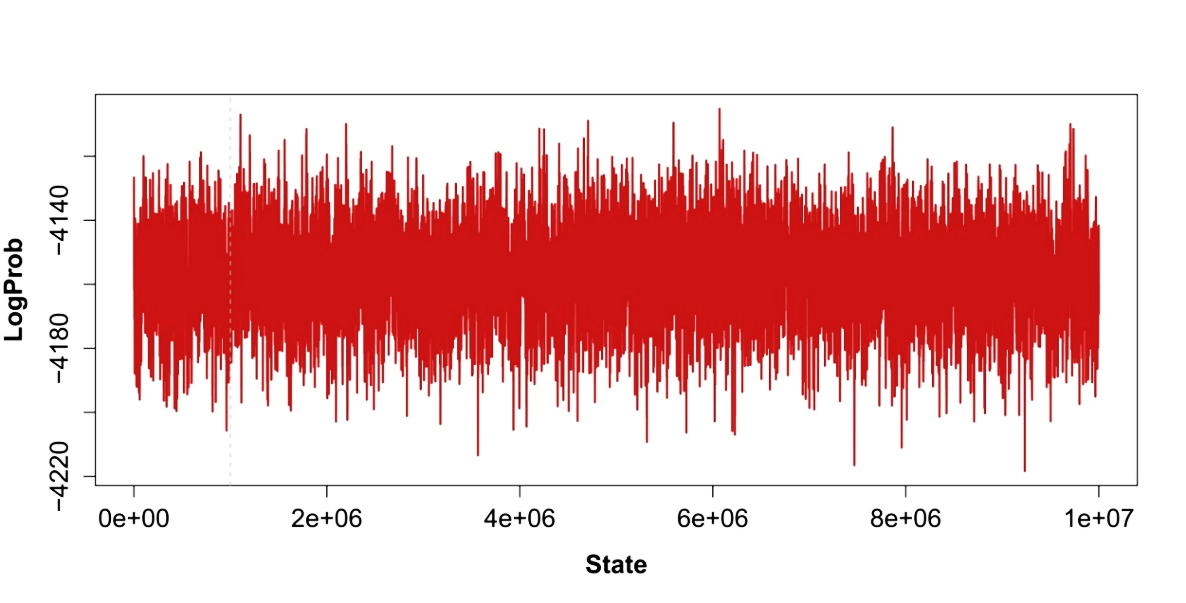


**Figure S4.** Convergence of MCMC chain with the highest deviance criterion indicated by TRACER

**Appendix S5**. Total and effective population size


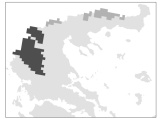

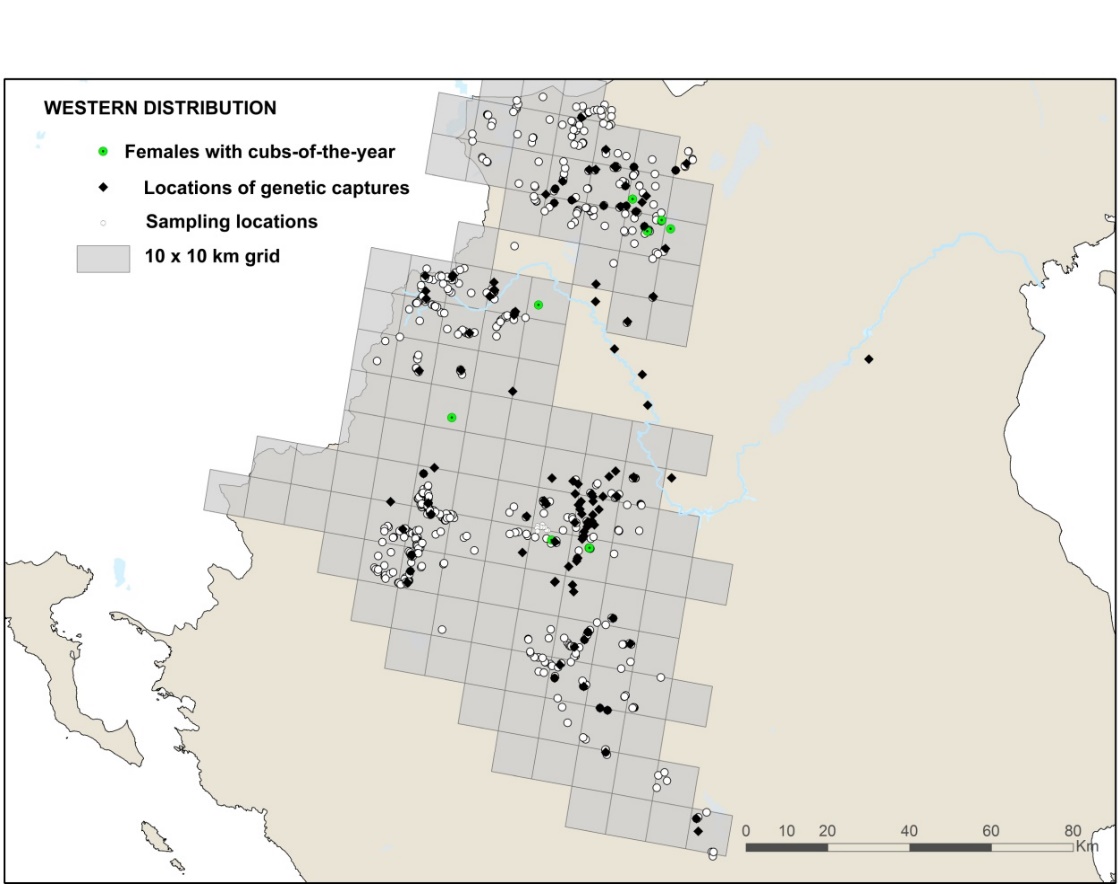


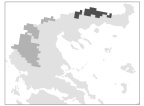
**
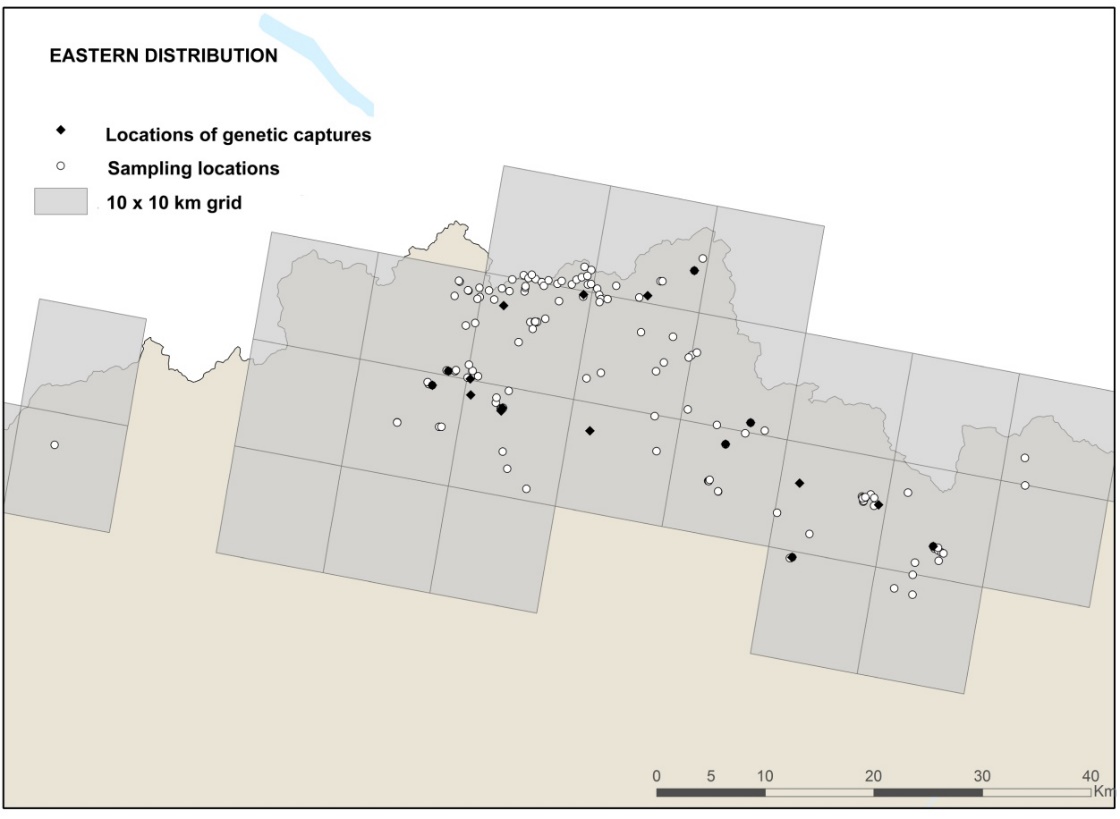
**

**Fig. S5.1** Distribution of observations of females with cubs-of-the-year, noninvasive sampling locations and genetic re-captures of individual brown bears used to estimate the population size using a capture-recapture approach for the western (top) and eastern (bottom) distributions, over a 10 x 10 km grid.

**Figure S5.2.** Capture frequencies of 143 living individual brown bears (blue bars, **Peristeri** n = 29; green bars, **Pindos** n = 92; red bars, **Rhodope** n = 22) used to estimate abundance in CAPWIRE. Two capture histories were derived by applying a minimum pairwise distance between two or more captures of the same individual of 500 m (top) and 1000 m (bottom).

**
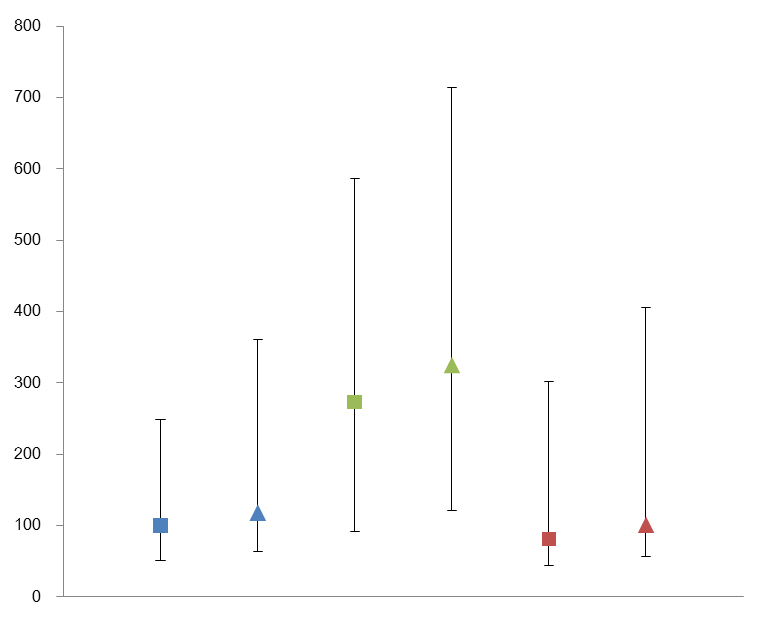
**

N_C_

**Peristeri**

**Pindos**

**Rhodope**

**Figure S5.3.** Population size estimates *N_C_* (± 95% CI) for each genetic brown bear subpopulation in Greece computed by CAPWIRE TRIM for two capture histories based on a fixed minimum pairwise distance between two captures (rectangles ≤ 500 m; triangles ≤ 1000 m).

**Appendix S6**. Mitochondrial DNA amplification

**Table S6** Homologous sequences used for median joining network

| **Genbank accession nr.** | **Study** | **Notation in Figure 3** |
| --- | --- | --- |
| AJ809334 | Hofreiter et al. 2004 | Aut |
| KJ638591 | Frosch et al. 2014 | Bgr1 |
| KJ638593 | Frosch et al. 2014 | Bgr3 |
| KJ638594 | Frosch et al. 2014 | Bgr4 |
| KJ638595 | Frosch et al. 2014 | Bgr5 |
| KJ638596 | Frosch et al. 2014 | Bgr6 |
| KJ638597 | Frosch et al. 2014 | Bgr7 |
| X75864 | Taberlet & Bouvet 1994 | Bgr8 |
| X75867 | Taberlet & Bouvet 1994 | Cro1 |
| HQ602652 | Kocijan et al. 2011 | Cro2 |
| AM411397 | Calvignac et al. 2008 | Dza |
| AM411398 | Calvignac et al. 2008 | Dza |
| GU320765 | Garcia et al. (unpublished) | Esp1 |
| X75865 | Taberlet & Bouvet 1994 | Esp1 |
| X75866 | Taberlet & Bouvet 1994 | Esp1 |
| GU186055 | Garcia et al. (unpublished) | Esp10 |
| GU186057 | Garcia et al. (unpublished) | Esp11 |
| GU186052 | Garcia et al. (unpublished) | Esp12 |
| GU186060 | Garcia et al. (unpublished) | Esp13 |
| GU186058 | Garcia et al. (unpublished) | Esp14 |
| GU186049 | Garcia et al. (unpublished) | Esp15 |
| GU186059 | Garcia et al. (unpublished) | Esp16 |
| GU186062 | Garcia et al. (unpublished) | Esp17 |
| GU186063 | Garcia et al. (unpublished) | Esp2 |
| GU186061 | Garcia et al. (unpublished) | Esp3 |
| GU186054 | Garcia et al. (unpublished) | Esp4 |
| GU186050 | Garcia et al. (unpublished) | Esp5 |
| GU186047 | Garcia et al. (unpublished) | Esp6 |
| GU186056 | Garcia et al. (unpublished) | Esp7 |
| GU186048 | Garcia et al. (unpublished) | Esp8 |
| GU186051 | Garcia et al. (unpublished) | Esp9 |
| GU186053 | Garcia et al. (unpublished) | Esp9 |
| HQ685901 | Keis et al. 2013 | Est |
| EU497665 | Bon et al. 2008 | Fra1 |
| X75878 | Taberlet & Bouvet 1994 | Fra2 |
| X75870 | Taberlet & Bouvet 1994 | Grc1 |
| AP012591 | Hirata et al. 2013 | Bgr9 (H21) |
| KJ638592 | Frosch et al. 2014 | Bgr2 (H21) |
| GR28 (Identical to AP012591 & KJ638592) | This study | H21 |
| GR33 (Identical to AP012591 & KJ638592) | This study | H21 |
| GR35 (Identical to AP012591 & KJ638592) | This study | H21 |
| GR36 (Identical to AP012591 & KJ638592) | This study | H21 |
| GR39a (Identical to AP012591 & KJ638592) | This study | H21 |
| GR40 (Identical to AP012591 & KJ638592) | This study | H21 |
| GR41 (Identical to AP012591 & KJ638592) | This study | H21 |
| GR42 (Identical to AP012591 & KJ638592) | This study | H21 |
| GR43 (Identical to AP012591 & KJ638592) | This study | H21 |
| GR10 (KR021974) | This study | H31 |
| GR12 (KR021974) | This study | H31 |
| GR14 (KR021974) | This study | H31 |
| GR15 (KR021974) | This study | H31 |
| GR16 (KR021974) | This study | H31 |
| GR17 (KR021974) | This study | H31 |
| GR18 (KR021974) | This study | H31 |
| GR19 (KR021974) | This study | H31 |
| GR20 (KR021974) | This study | H31 |
| GR44 (KR021974) | This study | H31 |
| GR46 (KR021974) | This study | H31 |
| GR47 (KR021974) | This study | H31 |
| GR48 (KR021974) | This study | H31 |
| GR49 (KR021974) | This study | H31 |
| GR5 (KR021974) | This study | H31 |
| GR50 (KR021974) | This study | H31 |
| GR51 (KR021974) | This study | H31 |
| GR52 (KR021974) | This study | H31 |
| GR53 (KR021974) | This study | H31 |
| GR54 (KR021974) | This study | H31 |
| GR55 (KR021974) | This study | H31 |
| GR56 (KR021974) | This study | H31 |
| GR57 (KR021974) | This study | H31 |
| GR58 (KR021974) | This study | H31 |
| GR59 (KR021974) | This study | H31 |
| GR6 (KR021974) | This study | H31 |
| GR68 (KR021974) | This study | H31 |
| GR69 (KR021974) | This study | H31 |
| GR70 (KR021974) | This study | H31 |
| GR71 (KR021974) | This study | H31 |
| GR72 (KR021974) | This study | H31 |
| GR74 (KR021974) | This study | H31 |
|  |  |  |
| GR77 (KR021974) | This study | H31 |
| GR78 (KR021974) | This study | H31 |
| GR79 (KR021974) | This study | H31 |
| GR8 (KR021974) | This study | H31 |
| GR84 (KR021974) | This study | H31 |
| GR85 (KR021974) | This study | H31 |
|  |  |  |
| GR89 (KR021974) | This study | H31 |
| GR3 (KR021975) | This study | H32 |
| GR4 (KR021975) | This study | H32 |
| GR60 (KR021975) | This study | H32 |
| GR63 (KR021975) | This study | H32 |
| GR64 (KR021975) | This study | H32 |
| GR67 (KR021975) | This study | H32 |
| GR9 (KR021975) | This study | H32 |
| GR1 (Identical to HE657212) | This study | H33 |
| GR73 (Identical to HE657212) | This study | H33 |
| GR82 (Identical to HE657212) | This study | H33 |
| HE657212 | Hailer et al. 2012 | Swe1 (H33) |
| FN292974 | Calvignac et al. 2009 | Irn |
| FN292975 | Calvignac et al. 2009 | Irn |
| X75862 | Taberlet & Bouvet 1994 | Ita1 |
| FN292971 | Calvignac et al. 2009 | Lbn |
| JQ823244 | Bray et al. 2013 | Nor1 |
| X75871 | Taberlet & Bouvet 1994 | Nor2 |
| HE657200 | Hailer et al. 2012 | Rom1 |
| HE657213 | Hailer et al. 2012 | Rom1 |
| HE657214 | Hailer et al. 2012 | Rom2 |
| HE657199 | Hailer et al. 2012 | Rom3 |
| KF545623 | Salomashkina et al. 2014 | Rus |
| KF545624 | Salomashkina et al. 2014 | Rus |
| KF545625 | Salomashkina et al. 2014 | Rus |
| X75868 | Taberlet & Bouvet 1994 | Swe2 |
| X75877 | Taberlet & Bouvet 1994 | Svn |
| KT438634 | Çilingir et al. 2016 | Tur1 |
| KT438636 | Çilingir et al. 2016 | Tur10 |
| KT438639 | Çilingir et al. 2016 | Tur2 |
| KT438638 | Çilingir et al. 2016 | Tur3 |
| KT438621 | Çilingir et al. 2016 | Tur4 |
| KT438622 | Çilingir et al. 2016 | Tur4 |
| KT438623 | Çilingir et al. 2016 | Tur4 |
| KT438624 | Çilingir et al. 2016 | Tur4 |
| KT438625 | Çilingir et al. 2016 | Tur4 |
| KT438626 | Çilingir et al. 2016 | Tur4 |
| KT438627 | Çilingir et al. 2016 | Tur4 |
| KT438628 | Çilingir et al. 2016 | Tur4 |
| KT438629 | Çilingir et al. 2016 | Tur4 |
| KT438630 | Çilingir et al. 2016 | Tur4 |
| KT438631 | Çilingir et al. 2016 | Tur4 |
| KT438632 | Çilingir et al. 2016 | Tur4 |
| KT438633 | Çilingir et al. 2016 | Tur4 |
| KT438643 | Çilingir et al. 2016 | Tur4 |
| KT438644 | Çilingir et al. 2016 | Tur4 |
| KT438645 | Çilingir et al. 2016 | Tur4 |
| KT438646 | Çilingir et al. 2016 | Tur4 |
| KT438647 | Çilingir et al. 2016 | Tur4 |
| KT438648 | Çilingir et al. 2016 | Tur4 |
| KT438649 | Çilingir et al. 2016 | Tur4 |
| KT438635 | Çilingir et al. 2016 | Tur5 |
| KT438650 | Çilingir et al. 2016 | Tur5 |
| KT438652 | Çilingir et al. 2016 | Tur5 |
| KT438653 | Çilingir et al. 2016 | Tur5 |
| KT438640 | Çilingir et al. 2016 | Tur6 |
| KT438641 | Çilingir et al. 2016 | Tur6 |
| KT438642 | Çilingir et al. 2016 | Tur6 |
| KT438651 | Çilingir et al. 2016 | Tur7 |
| KT438654 | Çilingir et al. 2016 | Tur7 |
| KT438655 | Çilingir et al. 2016 | Tur8 |
| KT438637 | Çilingir et al. 2016 | Tur9 |

**Appendix S7**. Genetic diversity across European populations

**Table S7** Genetic variability comparison between the studied subpopulations (bold) and other brown bear populations. (*A*) Mean number of alleles per locus, (*H_E_*) expected heterozygosity and (*N*) sample size for each study.

| **Population** | **Country** | **N** | **A** | **H_E_** | **Study** |
| --- | --- | --- | --- | --- | --- |
| Scandinavian | Norway NE | 75 | N/A | 0.78 | Eiken *et al.* 2009 |
|  | Norway NW | 26 | N/A | 0.69 | Eiken *et al.* 2009 |
|  | Norway M | 43 | N/A | 0.74 | Eiken *et al.* 2009 |
|  | Norway S | 62 | N/A | 0.72 | Eiken *et al.* 2009 |
|  | Scandinavia NN | 29 | 5.6 | 0.66 | Waits *et al.* 2000 |
|  | Scandinavia NS | 108 | 6.2 | 0.66 | Waits *et al.* 2000 |
|  | Scandinavia M | 88 | 5.9 | 0.66 | Waits *et al.* 2000 |
|  | Scandinavia S | 155 | 5.4 | 0.66 | Waits *et al.* 2000 |
| Karelian | Finland N (Pasvik) | 41 | 7.9 | 0.77 | Kopatz *et al.* 2012 |
|  | Finland S (Karelia) | 78 | 9.7 | 0.81 | Kopatz *et al.* 2012 |
| Carpathian | Romania | 109 | 8.5 | 0.80 | Straka *et al.* 2012 |
|  | Slovakia N | 71 | 6.1 | 0.71 | Straka *et al.* 2012 |
|  | Slovakia C | 96 | 6.0 | 0.70 | Straka *et al.* 2012 |
|  | Slovakia E | 16 | 5.2 | 0.65 | Straka *et al.* 2012 |
| East Balkan | Bulgaria | 136 | 8.9 | 0.74 | Frosch *et al.* 2014 |
|  | **Greece** **(Rhodope)** | 22 | 6.1 | **0.73** | **This study** |
| Dinaric-Pindos | Slovenia | 513 | 6.7 | 0.73 | Skrbinsek *et al.* 2012 |
|  | Croatia | 156 | 7.6 | 0.74 | Kocijan *et al.* 2011 |
|  | **Greece** **(Peristeri)** | 30 | 5.6 | **0.69** | **This study** |
|  | **Greece** **(Pindos)** | 97 | 5.2 | **0.64** | **This study** |
| Cantabrian (W) | Spain | 39 | 3.3 | 0.45 | Perez *et al.* 2009 |
| Cantabrian (E) | Spain | 8 | 1.7 | 0.25 | Perez *et al.* 2009 |
| Apennines | Italy | 17 | 2.4 | 0.46 | Zachos *et al.* 2008 |

Eiken HG, Andreassen RJ, Kopatz A, Bjervamoen SG, Wartiainen I, Tobiassen C, *et al*. (2009) Population data for 12 STR loci in Northern European brown bear (Ursus arctos) and application of DNA profiles for forensic casework*. Forensic Science International: Genetics Supplement Series*, *2*, 273–4.

Frosch C, Dutsov A, Zlatanova D *et al*. (2014) Noninvasive genetic assessment of brown bear population structure in Bulgarian mountain regions. *Biology*, 79, 268–276.

Kocijan I, Galov A, Ćetković H, Kusak J, Gomerčić T, Huber Ð (2011) Genetic diversity of Dinaric brown bears (*Ursus arctos*) in Croatia with implications for bear conservation in Europe. *Mammian Biology*, 76, 615–621.

Kopatz A, Eiken HG, Hagen S *et al*. (2012) Connectivity and population subdivision at the fringe of a large brown bear (*Ursus arctos*) population in North Western Europe. *Conservation Genetics*, 13, 681–692.

Perez T, Vazquez F, Naves J *et al*. (2009) Non-invasive genetic study of the endangered Cantabrian brown bear (*Ursus arctos*). *Conservation Genetics*, 10, 291–301.

Skrbinšek T, Jelencic M, Waits LP, Potocnik H, Kos I, Trontelj P (2012) Using a reference population yardstick to calibrate and compare genetic diversity reported in different studies: An example from the brown bear. *Heredity*, 109, 299–305.

Straka M, Paule L, Ionescu O, Stofik J, Adamec M (2012) Microsatellite diversity and structure of Carpathian brown bears (*Ursus arctos*): consequences of human caused fragmentation. *Conservation Genetics*, 13, 153–164.

Waits LP, Taberlet P, Swenson JE, Sandegren F, Franzen R (2000) Nuclear DNA microsatellite analysis of genetic diversity and gene flow in the Scandinavian brown bear (*Ursus arctos*). *Molecular Ecology*, 9, 421–431.

Zachos FE, Otto M, Unici R, Lorenzini R, Hartl GB (2008). Evidence of a phylogeographic break in the Romanian brown bear (*Ursus arctos*) population from the Carpathians. *Mamm Biol* 73: 93–101.
